# Recognizing and Mitigating the Effects of Medication on Heat-Related Illness in Older Adults: A Scoping Review

**DOI:** 10.3390/pharmacy14030074

**Published:** 2026-05-17

**Authors:** Lily M. Tews, Daniel T. Abazia, Hayley Blackburn, Kiri Carmody, Mary Barna Bridgeman

**Affiliations:** 1 Ernest Mario School of Pharmacy, Rutgers, The State University of New Jersey, Piscataway, NJ 08854, USA; lily.tews@rutgers.edu (L.M.T.); dabazia@pharmacy.rutgers.edu (D.T.A.); 2 Skaggs School of Pharmacy, University of Montana, Missoula, MT 59812, USA; hayley.blackburn@mso.umt.edu; 3 Skaggs School of Pharmacy & Pharmaceutical Sciences, University of Colorado, Aurora, CO 80045, USA; kiri.carmody@cuanschutz.edu

**Keywords:** heat, heat-related illness, thermal stress, older adults

## Abstract

Heat waves have intensified since the 1960s, leaving older adults uniquely susceptible to heat-related illnesses, including hyperthermia and fluid-electrolyte imbalances. While clinicians recognize that certain medications increase heat vulnerability, the specific interplay between drug use and patient characteristics remains unclear. This scoping review, following the Joanna Briggs Institute framework for scoping reviews and the Preferred Reporting Items for Systematic Reviews and Meta-Analyses extension for Scoping Reviews (PRISMA-ScR) guidelines, investigated the risk of heat-related illness associated with medication use in older adults to identify research gaps. Investigators queried four databases for English-language primary literature (2000–2025) based on predefined Population, Concept, and Context criteria. Additionally, a grey literature search mapped existing United States (U.S.) mitigation strategies. Two reviewers independently screened studies via Covidence, and one extracted data. Results included 61 primary studies and 41 grey literature sources. While epidemiological data confirm higher heat-related morbidity and mortality in older populations, few experimental studies evaluate medication’s specific role. Despite many public health efforts, specific, evidence-based guidance on managing drug-heat interactions is limited. Diuretics, angiotensin-converting enzyme (ACE) inhibitors, angiotensin receptor blockers (ARBs), anticholinergics, and antipsychotics were the medication classes most frequently associated with heat-related illness. This review underscores a critical need for research into the confluence of age, multimorbidity, and polypharmacy to inform future clinical mitigation and protect vulnerable populations.

## 1. Introduction

Climate change has been recognized by the World Health Organization as a fundamental health crisis, following 2024’s record-setting global temperatures [1,2,3,4]. As one of the most defining health issues of our time, the challenges posed by climate change permeate health disciplines and necessitate an interprofessional response to mitigation and management. Climate change medicine, also known as climate medicine, is a rapidly developing field that examines the interconnection between climate and human health, encompassing implications for health system sustainability practices, public health initiatives, and clinical care [5].

One focus of climate medicine is heat-related illness, which represents a direct consequence of high environmental temperatures on health. The United States (U.S.) Environmental Protection Agency (EPA) published characteristics of heat waves in the U.S. from 1961 to 2023; according to this data, heat waves have steadily increased in frequency, intensity, and duration during this time [6]. Heat-related illness encompasses a spectrum of disorders, from mild heat rash and cramps to potentially life-threatening heat exhaustion and heat stroke, that occur when an individual is unable to adapt to high temperatures [7]. Aside from these examples of heat injury, dehydration and electrolyte imbalance are two related conditions that tend to worsen during warm weather. Dehydration, or the net loss of fluid due to decreased consumption and/or increased loss, may result in electrolyte imbalances, such as disturbances in serum sodium levels (e.g., hyponatremia and hypernatremia) or potassium levels (e.g., hypokalemia and hyperkalemia). Underlying chronic illness, medication use, and age are known risk factors for dehydration and electrolyte abnormalities [8].

Older adults are uniquely susceptible to heat due to their vulnerability toward heat-related illness, including hyperthermia due to thermoregulatory impairment (e.g., reduced evaporative cooling secondary to reduced sweat gland output, reduced cutaneous vasodilation), dehydration, and electrolyte abnormalities [9,10,11]. Further, medication use may contribute to heat sensitization and heat illness, especially among older adults [12]. Significant evidence for an increase in core temperature and thermoregulatory impairment is likely with strong anticholinergic medications, adrenaline, anti-Parkinson’s agents, and non-selective beta blockers [13]; a need for further investigation and quantification of the impact of all potential medications believed to increase risks during heat stress in vulnerable older adults remains.

It is generally known that medication use is a key factor in the susceptibility of older adults to heat-related illness [14]. Both climate change and high rates of prescription drug use accentuate the need for additional research. According to a Kaiser Family Foundation Health Tracking Poll, 89% of adults aged 65 and older reported taking prescription medication in 2019, with 54% having been prescribed four or more medications [15]. An improved understanding of the exact role of medications in heat-related illness risk, including interactions with age-related thermoregulatory decline and existing chronic conditions, is therefore necessary to guide clinicians and caregivers in improving health outcomes for older adults.

The objective of this scoping review was to investigate the current literature regarding the heightened risk of heat-related illness in older adults due to medication use and age-related vulnerabilities. A scoping review methodology was adopted for this project to inventory the breadth of evidence, identify key concepts and clarify research and practice gaps. Specifically, the following research questions were posed: (1) What evidence exists for heat sensitivity in older adults; (2) which drug classes appear to possess the highest risk of thermoregulatory impairment, electrolyte abnormalities, and dehydration for older adults during heat waves; and (3) what strategies have already been implemented to mitigate the impact of heat-related illness in older adults? By collating the current literature and identifying knowledge gaps, this review aims to identify areas of future research that are particularly relevant to rising global temperatures.

## 2. Materials and Methods

### 2.1. Inclusion Criteria

This scoping review was conducted according to the Joanna Briggs Institute (JBI) framework for scoping reviews, which is congruent with the Preferred Reporting Items for Systematic reviews and Meta-Analyses extension for Scoping Reviews (PRISMA-ScR) guidelines [16,17]. Reviewers developed and adhered to an a priori protocol. Sources were included in the scoping review based on the Population, Concept, and Context (PCC) framework, as they pertained to the research questions. The population was older adults aged 65 and older. The concept of this review consists of two related phenomena of interest: heat sensitivity associated with age and heat-related illness associated with medication use. Only sources examining these phenomena in the context of high environmental temperatures were included. Primary studies and case reports published in English after the year 2000 were included. Previous reviews and commentaries were excluded. Grey literature was included to address the third research question; however, the search for existing mitigation efforts was limited to the United States for feasibility purposes. The format of the grey literature selected was diverse and included the U.S. Food and Drug Administration (FDA) Label (FDALabel) database, an archive of medication labeling documents submitted by manufacturers (discussed below). It should be noted that the U.S. FDALabel database search was not limited to a specific population; instead, investigators scrutinized medication structured product labeling (SPL) for medications likely to have specific heat-related concerns in older adults [15].

### 2.2. Search Strategy

An experienced health sciences librarian assisted in developing the search strategy. Investigators queried the databases PubMed, Embase, Scopus, and Web of Science for the primary literature. Advanced queries contained four categories consisting of relevant terms and controlled vocabulary: age, temperature, heat-related illness, and medication use. The complete search strategy for each database is provided in File S1. The search spanned from January 2000 to June 2025. For the grey literature search, investigators used simple keywords such as “heat illness” and “older adults” to manually search the U.S. Centers for Disease Control and Prevention (CDC) Climate and Health website and the National Institute on Aging (NIA) website. The U.S. FDALabel database was queried using the following filters: Human Prescription Drug: ANDA, NDA, or ANDA Authorized generic; “heat” within Warnings and Precautions; and Inhalation, Ophthalmic, Oral, Subcutaneous, Topical, and Transdermal. The most recent version of the medication SPL document was included.

### 2.3. Screening

Investigators used Covidence systematic review management software (Veritas Health Innovation Ltd., Melbourne, Australia. Available at www.covidence.org.) for importation, de-duplication, and screening of references. After importing and merging database search results into Covidence, one reviewer manually screened the duplicates identified by Covidence to confirm their exclusion. Two reviewers independently screened titles and abstracts for eligibility, and discrepancies were resolved by discussion of conflicting references until consensus was reached. Two reviewers independently selected studies for inclusion during full-text screening. Although the protocol stated that conflicts would again be resolved by consensus, a third reviewer made the final screening decision. Reasons for exclusion at this stage were recorded.

For the selection of the grey literature, one reviewer manually searched the specified websites and selected webpages, documents, and other available literature for inclusion in the review. The grey literature sources were added to the PRISMA flow diagram after selection and approval by a second reviewer. The protocol for the U.S. FDALabel database was slightly modified: one SPL document was retrieved for each drug and dosage form identified by the search, but some documents were excluded after further screening. Due to the similarity in labeling across different dosage forms of the same drug (e.g., aripiprazole tablet versus aripiprazole solution), only the SPL for one dosage form was ultimately included in the review. Additionally, some drugs included “heat” in “Warnings and Precautions” sections beyond the scope of this review; for example, there were warnings about Neuroleptic Malignant Syndrome, protection from heat during medication storage, and increased absorption of a transdermal patch due to external heat sources. Investigators chose to include only documents with information on heat-related illness in this review.

### 2.4. Data Extraction

One reviewer used the data extraction tool available in Table S1 to comprehensively extract data from the included literature, with validation by other reviewers. Although the protocol indicated that this table would be used for all grey literature sources, it was not used for documents retrieved from the U.S. FDALabel database. Instead, the reviewer transcribed relevant statements from the “Warnings and Precautions” and “Patient Counseling Information” sections of each document, along with the generic drug name and class.

### 2.5. Analysis

Basic information, such as year of publication, study type, and country of origin, was recorded for all primary studies and presented in tabular or graphical form. Additionally, investigators analyzed the frequency at which the following concepts were present: age-related heat sensitivity (including the factors that are measured or discussed, such as chronic illness); medication use during extreme heat (including the risks that are measured or discussed, such as impaired sweating); drug or drug classes implicated in heightened heat risk; and efforts to mitigate heat-related illness in older adults. The frequency of pertinent concepts is presented as a table and a detailed narrative summary.

The grey literature required a separate approach to analysis and presentation. The origin, format, and intended audience were recorded for all resources included in the review. Investigators examined the information in each resource, with particular attention to any mentions of medication use. The basic characteristics of the included resources were tabulated, and a narrative description of the grey literature summarizing existing efforts in the U.S. to mitigate the impact of heat-related illness among older adults was developed. Investigators also documented which drugs appeared in the U.S. FDALabel database search to identify strengths and weaknesses in current regulatory information.

## 3. Results

### 3.1. Search Results

**Figure 1:**
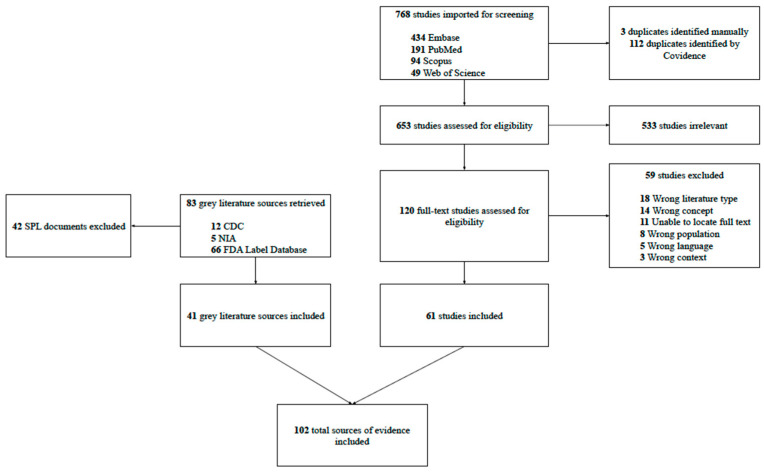
PRISMA flow diagram of the selection of eligible studies. PRISMA, Preferred Reporting Items for Systematic Reviews, depicting the literature search decision process.

A total of 768 studies were imported for screening, with 112 duplicates identified by Covidence, and 3 duplicates later identified manually by reviewers. During title and abstract screening, 653 studies were assessed for eligibility, and 533 were excluded. During full-text screening, 120 studies were assessed for eligibility, and 59 were excluded for the following reasons: 18 for Wrong Literature Type, 14 Wrong Concept, 11 Unable to Local Full Text, 8 Wrong Population, 5 Wrong Language, and 3 Wrong Context. A total of 83 grey literature sources were retrieved (12 from the U.S. CDC’s Climate and Health website, 5 from the NIA website, and 66 from the FDA Label database). Forty-two SPL documents were excluded upon further review, leaving a total of 24 SPL documents and 41 total grey literature sources included in this review. The search decision process is summarized in Figure 1.

To justify the inclusion of each primary and grey literature source, a Table of Included Sources of Evidence is provided in Table S2. A modified version of the Charting Table for Data Extraction, this figure clearly outlines the key findings and relevance of each source to the research questions and helps to guide reviewers in constructing a narrative summary of the literature. Additionally, basic characteristics of both the included primary studies and grey literature sources are summarized in Table S3 and Table S4, respectively.

### 3.2. What Evidence Exists for Heat Sensitivity in Older Adults?

The outcome of interest in 19 studies was hyperthermia-related conditions (e.g., heat stroke, heat exhaustion, heat syncope). Multiple observational studies consistently identify age as a primary risk factor; for instance, individuals over 65 represented a disproportionate share of heat-related hospital admissions and exhibited increased mortality risk during ‘on alert’ heat periods [18,19,20]. Likewise, a 2014 retrospective observational study in Georgia found that the odds of admission versus Emergency Department (ED) discharge increased with age, with the highest odds ratio observed in patients aged 80 and older and in those with certain comorbidities [21]. Two cohort studies also assessed hyperthermia-related conditions. One of these studies analyzed drug-associated hyperthermia in particular; investigators determined that 51.8% of patients presenting with hyperthermia were older than 60 years, and the older age group had both a higher mean Charlson Comorbidity Index and number of concomitant medications [22]. The other cohort study examined prognostic factors in non-exertional heat stroke; these included living in an institution, age greater than 80 years, cardiac disease, and cancer. Dependence was not a prognostic factor in this study, as 35% of non-survivors were autonomous prior to admission [23].

While case reports suggest a link between aging and heat vulnerability, these anecdotal findings primarily serve to illustrate potential risks rather than establish population-wide prevalence [24,25,26]. A case series of multiple heat-related fatalities from Wisconsin in 2012 identified age, cardiovascular disease, and mental illness as risk factors [27]. A 2020 case series analysis indicated that the study population (i.e., older Medicare beneficiaries with chronic comorbidities taking heat-sensitizing medications) was at increased risk of heat-related hospitalization, even when heat levels are not extreme [28].

Four case-control studies provide additional evidence of heat sensitivity in older adults. One study from 2014 and its 2015 extension found that patients aged 60 and older appeared to be most vulnerable to syncope, and that cases were more frequent in the summer months, a phenomenon the authors called “summer syncope syndrome” [29,30]. In a matched case-control study investigating psychotropic drug use and heat-related hospital admissions, the mean age of patients presenting with heat-related conditions was 83 years, and 32% were aged 90 and older [31]. In contrast, a different case-control study looked at risk factors for hyperthermia mortality in ED patients and found that the mean age of cases was just 56 years (statistically significant risk factors included past ED utilization for alcohol use, receiving Medicare benefits, or having no insurance) [32]. A retrospective risk-factor analysis published in 2006 examined heat stroke patients admitted to intensive care units in France and found that the mean age of these patients was approximately 67 years [33]. In a case-only study conducted in New South Wales, men aged 75 and older were most likely to be hospitalized with a primary diagnosis of heat-related illness, along with individuals aged 65 and older with underlying cerebrovascular disease [34]. One cross-sectional study revealed age as a risk factor for heat-related illness; in a population of behavioral health disorder (BHD) hospitalizations, increasing age, dementia, and schizophrenia were among the risk factors for concurrent BHD and heat-related illness [35]. A 2016 sequence symmetry analysis investigated hospital admissions for both heat illnesses and dehydration after the initiation of medications, and, notably, the median age of patients in this study was 85 years [36].

Several analyses examined the susceptibility of older adults to dehydration and electrolyte imbalances during extreme heat, including many cohort studies. One of these, an extension to the 2010 study, found that dementia and institutional living were independently associated with hypernatremia, and that heatstroke severity score was associated with hyponatremia among hyperthermic patients. The mean age of admitted hyperthermic patients was 82 [37]. Similarly, the mean age of patients diagnosed with thiazide-induced hyponatremia in an earlier study was 76 years [38]. Two cross-sectional studies confirmed both the predisposition of older adults to hyponatremia and its seasonality; one of them found a 1.2% increased risk per degree Celsius [39,40]. A different cross-sectional study found age greater than 80 years to be an independent predictor of mortality in general during heat waves but did not find a significant association between extreme heat and electrolyte disorders in older adults [41]. Conversely, one study involving hemodialysis patients in Greece discovered a relationship between hyperkalemia and season, but not between hyperkalemia and age [42]. The role of environmental temperature in renal function is a related inquiry to these electrolyte disorders. One cohort study assessed the effects of both high ambient temperature on antihypertensive treatment and found that the negative effect of temperature on kidney function was most pronounced in patients older than 75 years [43]. Another cohort study concluded that extensive fluid loss in the summer may lead to mild renal impairment in hospitalized older adults, but compensatory physiological mechanisms may make this a clinically insignificant finding [44].

Four studies used adverse drug reaction (ADR) reports to address research questions. In a 2017 case-crossover conducted in Sweden, the median age of patients with reported drug-induced hyponatremia was 80 years [44]. A prospective evaluation in Spain likewise found a significant association between drug-induced severe hyponatremia and age [45]. In a population of patients aged 70 years and older during summer 2003 in France, a total of 68 heat-related ADRs occurred (typically metabolic or neuropsychiatric in nature), with women and patients aged 80 years and older more likely to experience these occurrences [46]. Another retrospective study also investigated patients older than 70 years in France; significantly more serious ADRs were heat-related in 2003 (with a heat wave) than in 2006 (reference period without a heat wave) [47].

In addition to studies investigating specific health outcomes in older adults, a variety of publications report all-cause morbidity or mortality associated with extreme heat. Five descriptive reports were included in this review, all of which analyze the same European heatwave that occurred in summer 2003. In the Netherlands, researchers found a strong association between increasing age and excess mortality with a pronounced impact among institutionalized older adults [48]. In England and Wales, excess mortality during the heatwave was greatest in the 75 and older age group [49]. In four Italian cities, excess mortality was the greatest in the 75–84 years and 85 years and older age groups, respectively [50]. Likewise, in Spain, excess mortality was observed in only the 74–84 and 85 years and older groups [51]. In France, excess mortality was also the greatest among older adults, with 70% in the 75–94 years old group and 20% in the 94 years and older group. Excess mortality due to heat-related disease reached 1860% during a period of extreme heat in France [52].

A cohort study in Italy found that age, being unmarried, and certain comorbidities were significant effect modifiers for mortality during heat waves, though the cause of death was not analyzed [53]. An earlier cohort study in the U.S. examined emergency medical service calls during heatwaves; interestingly, the 65–84 year and 85 years and older age groups were not associated with the greatest risk according to study findings [54]. Four time-series analyses explored all-cause morbidity and mortality during extreme heat. One of these studies also examined emergency medical service calls, and the results indicate that patients aged 75 years and older comprised the largest proportion of health-related emergency calls, had lower thresholds at which high temperatures affected emergency calls, and were more sensitive to higher maximum temperatures [55]. In another study, the 65–74 year and 75 years and older age groups in Australia were especially vulnerable to heat and had higher total, respiratory, and cardiovascular ambulance attendances [56]. Two additional time-series analyses found an association between mortality and age during high-temperature periods [57,58].

A case-crossover study investigated the effect of temperature on heat-related General Practitioner (GP) consultations among patients with type 2 diabetes. While patients aged 65 years and older appeared to be at a heightened risk for heat-related GP consultations, the effect was not statistically significant [59]. Among patients with congestive heart failure, increased maximum daily temperature was associated with increased daily mortality in a different study [60]. The univariate analysis of another case-crossover study suggests age, living alone, and use of community services as significant risk factors for heat-related hospitalization [61]. One cross-sectional study in Canada examined the prevalence of predefined risk factors for general heat-related health outcomes, with the most prevalent factors being cardiovascular medication, hypertension, living alone, cardiovascular disease, residing in an urban heat island, and needing assistance with daily activities [62]. Finally, a 2005 case-control study in France involved patients with and without autonomic failure (AF) who may experience orthostatic hypotension (OH). Although no significant association was found between age and OH-related events during the heatwave, the incidence of OH-related events was significantly higher during the heatwave in AF patients than in controls, suggesting that AF may be an important comorbidity to consider in heat-related health outcomes [63]. Based on these findings, the evidence suggests an increase in heat sensitivity and vulnerability among older adults.

### 3.3. Which Drug Classes Appear to Possess the Highest Risk of Thermoregulatory Impairment, Electrolyte Abnormalities, and Dehydration for Older Adults During Heat Waves?

Table 1 summarizes the study findings on the heat-related effects of specific therapeutic and pharmacological classes. 

Although physiological mechanisms suggest that cardiovascular drugs could impair thermoregulation, empirical evidence remains conflicting. Conversely, classes such as diuretics, ACE inhibitors, and ARBs are more consistently associated with empirical evidence of collateral effects like dehydration and hyponatremia [43,66,67]. For instance, one study did not specify a particular drug class but found that the odds ratio for drug-induced hyponatremia increased with increasing temperature during the summer months [44]. Psychotropic medications are frequently hypothesized to impair thermoregulation, though some studies find that the risk of heat-related illness in patients with mental illness persists regardless of medication use, suggesting a complex interplay between drug effects and underlying pathology [31,68]. Moreover, medications with anticholinergic properties inhibit dissipation of heat and temperature regulation, thus increasing vulnerability of at-risk populations to heat-related harms, as suggested by several studies and case reports. It is notable that the concomitant use of medications in the treatment of multi-morbid health conditions in the older adult and polypharmacy may augment or exacerbate risk; specific evidence on the risks of polypharmacy on heat-related illness was not elucidated in this review.

### 3.4. What Strategies Have Already Been Implemented to Mitigate the Impact of Heat-Related Illness in Older Adults?

Recommended strategies to mitigate the impact of heat-related illness in older adults largely rely on physiologic reasoning and environmental data. Much of the existing information on the prevention and management of heat-related illness in older adults is found in the grey literature. This review found a lack of randomized, controlled trials evaluating the efficacy of different strategies for medication management and the prevention of heat-related illness in older adults, although data on general strategies for prevention of heat-related illness in older adults are available.

Old age is identified as a risk factor in three simple informative webpages retrieved from the U.S. CDC and intended for the public [14,73,74]. These pages highlight older adults as a population of particular concern for adverse outcomes due to heat exposure, with a specific webpage titled “Heat and Older Adults (Aged 65+)”, intended to educate older adults and caregivers on the risks of heat-related illness during extreme heat. This resource provides several preventative measures to take, including staying indoors with air conditioning as much as possible, not relying on fans alone when it is extremely hot outside, drinking plenty of water, avoiding use of the stove or oven, wearing appropriate clothing, taking cool showers or baths, avoiding strenuous activities, getting enough rest, and having someone check on older adults who are living alone. Prescription medication use is listed as a risk factor. Another resource from the U.S. EPA shared similar information on rising global temperatures, how heat affects health, and what people can do before and during an extreme heat event to minimize health impacts. Older adults are identified as a sensitive population, and medication use is briefly mentioned as a risk factor. In addition to an easy-to-follow overview of climate change and heat-related illness, this resource provides a list of “Do’s” and “Don’ts” to follow during an extreme heat event, as well as actions to take beforehand, including checking on older, sick, or disabled people, as well as those who live alone, during heat waves [75].

Multiple U.S. CDC and NIA webpages recognize the importance of staying informed about the weather in preventing heat-related illness [76,77,78,79,80,81,82,83,84,85,86,87]. The Heat & Health Tracker, an interactive tool developed by the U.S. CDC, allows users to enter their U.S. Postal Service Zone Improvement Plan (ZIP) code and view heat and health data for their area at any time [76]. The Heat & Health Tracker uses U.S National Weather Service HeatRisk categories to specify risk levels, sensitive populations, and actions to take under different heat conditions [77].

Additional U.S. CDC resources for healthcare professionals included in this review also provide tools for clinical use and information for clinicians. The “CHILL’D-Out” questionnaire, which stands for Cooling, Housing, Isolation and mobility, eLectricity, Learning, Drugs, and Outside, allows clinicians to assess risk factors and ultimately construct a Heat Action Plan with their patients [78]. The U.S. CDC’s “Quick Start Guide for Clinicians on Heat and Health” directs clinicians to assess patient risk factors using the CHILL’D-Out questionnaire, teach patients how to use the HeatRisk and Air Quality Index tools, and educate patients on further steps they can take by developing a Heat Action Plan [79]. Two other U.S. CDC resources provided a broad overview of the clinical significance of extreme heat and a concise outline of medications that may increase the risk of heat-related illness [80,81]. To ensure patient safety during hot weather, it is recommended that clinicians consider a range of strategies, including (1) adjusting medication dosage or frequency based on possible interactions with heat, particularly for older patients who may take multiple high-risk medications; (2) consider adjusting fluid restrictions, particularly for patients taking medications that may affect fluid-electrolyte balance; (3) have at-risk patients identify a point of contact to check on them during extreme heat, particularly older adults and those with altered cognition; and (4) educate patients on possible symptoms of drug interactions with heat, heat-related symptoms requiring emergency care, and a clear plan of what to do in such an emergency [81]. Additional resources from the NIA and content from a virtual research workshop provide similar information [82,83,84,85,86].

Literature related to U.S.-based public health planning was also included in this review. The U.S. CDC Climate-Ready States and Cities Initiative (CRSCI) provides examples of public health planning efforts across 16 states and 2 cities, including the creation of Climate and Health Adaptation Plans, which address adaptations to extreme heat in the long-term, and Heat Response Plans, which guide government agencies and partners through a coordinated emergency response during heat waves [87]. Components of these multi-faceted public health planning efforts include communication materials and education, heat health alert systems, cooling centers and water sites, home energy assistance and weatherization, surveillance of heat-related morbidity and mortality, assessments and analyses to investigate community-specific heat impacts, dissemination of climate and health vulnerability data, and partnerships with local community organizations. Within CRSCI efforts, older adults, populations with chronic medical conditions, and those living in social isolation were consistently identified as among the most at-risk groups requiring special consideration in public health planning [87].

The CRSCI technical report presents a variety of existing strategies that public health officials may implement to mitigate the effects of extreme heat in their own jurisdictions, with examples of success stories across the CRCSI programs, such as the creation of heat advisory thresholds, the Low Income Home Energy Assistance program (LIHEAP) to help low-income households pay for utilities or cooling devices, and the use of syndromic surveillance to determine heat illness burden, examine risk factors and populations, and create effective interventions Authors of this technical report acknowledge the mixed evidence and methodological challenges of evaluating the efficacy of individual components of a heat response plan and their impacts on health outcomes [88].

Information related to the prevention of heat-related illness is also included in the U.S. FDA SPL for many medications that may impair thermoregulation or otherwise increase the risk of heat-related illness. Of the 24 SPL included in the review, 12 (50.0%) were for antipsychotic drugs, 5 (20.8%) for anticholinergic drugs, and 2 (8.33%) for anticonvulsant drugs. One label each (4.17%) was found for an SGLT2 inhibitor, antipsychotic and SSRI, antipsychotic and opioid antagonist, antiemetic and antipsychotic, and anorexiant. The provided information on patient counseling focused primarily on avoiding heat exposure, managing dehydration, self-monitoring, and when to contact a healthcare professional if signs of overheating or heat-related illness are observed. A complete list of these drugs, along with the information provided on each label, is presented in Table 2.

Although investigators intended for the primary literature to address the first two research questions, six studies were identified that focused exclusively on interventions to reduce the risk of heat-related illness. Dietary nitrate supplementation was one clinical intervention experimentally proven to be unsuccessful in providing patients with protection during heat stress [113]. In contrast, a 2019 experiment found that chronic statin treatment may improve reflex cutaneous vasodilatation during heat stress in hypercholesterolemic older adults [114], and a cohort study published in the same year discovered an increasing survival benefit of empiric potassium among furosemide users as daily maximum temperature increases, suggesting a possible intervention to reduce excess mortality during extreme heat due to potassium depletion [115]. Additionally, Australian researchers in 2021 used biophysical modeling to revise the temperature thresholds for recommending the use of electric fans. They did so with the decreased sweating ability of older adults with and without anticholinergic medication use in mind, changing the thresholds for the use of fans to <37.0 °C for older adults taking anticholinergic medications and <38.0 °C for healthy older adults, revised from the broad recommendation of fan use below temperatures of <35.0 °C only for all populations. Their model not only provided more specific guidance on fan use across different populations but also increased the number of days that electric fans can be used instead of carbon-intensive air conditioning [116]. One cross-sectional study assessed the implementation of the National Heat Plan in institutions for older adults in the Netherlands. While almost all care managers agreed with most of the cooling measures outlined in the heat plan, staff training and resident independence remained significant barriers to implementation of several measures, and only 41% of care managers considered consulting physicians on medication use to be “very important” [117]. Finally, the results of a 2010 survey found knowledge gaps among patients with diabetes living in hot climates and a need for more patient education. This study also found that patients with poor glycemic control may be at increased risk of dehydration during hot weather, underscoring the importance of recognizing patients with diabetes as a population of concern during extreme heat events [118].

## 4. Discussion

This scoping review suggests that older adults are vulnerable to heat-related illnesses. There is an abundance of observational studies indicating an association between advanced age and various outcomes, such as hyperthermia-related conditions, dehydration and electrolyte abnormalities, and all-cause morbidity or mortality during heat waves. Interestingly, many of these studies used data from a 2003 heat wave in Europe, including the five descriptive reports that documented excess mortality across several countries [49,50,51,52]. This heat wave was one of the ten deadliest natural disasters in Europe in the preceding 100 years, with significant consequences for agriculture, natural resources, and human health; older adults were disproportionately impacted [119]. Although the mechanisms underlying the increased heat sensitivity of older adults were not explored in most studies included in this review, epidemiological evidence has identified several risk factors, including age-related physiological changes (i.e., impaired renal function), comorbidities (i.e., cardiovascular conditions), social isolation, and dependence often experienced by older adults that may contribute to increased vulnerability to heat [19,23].

The literature summarized in Table 1 suggests an association between certain medication classes and heat-related health outcomes. Only two randomized controlled trials met the inclusion criteria for this review, and, notably, their results disprove their hypotheses that antihypertensives affect critical environmental limits and low-dose aspirin alters core or skin temperatures in warm-humid or hot-dry environments [64,65]. At the same time, numerous observational studies have cited diuretics, ACE inhibitors, and ARBs as medication-related risk factors in heat-related illness. Diuretics were the most frequently cited therapeutic class in the literature. Some studies have connected diuretics to hyperthermia-related conditions, such as heat stroke, while most have investigated their effect on fluid-electrolyte balance as a measure of renal function. Two studies found a significant association between drug-induced hyponatremia and environmental temperature, although a third found no association between diuretics and profound hyponatremia in warmer months [39,40,45]. The risk of dehydration or electrolyte abnormalities associated with certain antihypertensive medications is well-established, particularly regarding diuretic use [120]. Age as a predictor of renal impairment was also recognized in prior studies [121]. Thus, the potential correlation between these health outcomes and environmental temperature is pertinent to the present review and must be better understood to evaluate the multifaceted vulnerability of older adults to heat-related illness.

Antipsychotic medications appeared frequently in studies examining psychotropic drugs and heat-related illness. One matched case-control study found that antipsychotic and anxiolytic medications were associated with ED admissions for heatstroke or hyperthermia; in contrast, a self-controlled case series found that individuals with mental illness had a 40% to 60% increased risk of heat-related illness regardless of medication use [31,68]. The SPL for several antipsychotic medications warns of disruptions in one’s ability to reduce core body temperature during heat stress (see Table 2). Individuals with schizophrenia may additionally experience an intrinsic dysregulation of body temperature or be at risk for hyperthermia due to other physiological, social, or behavioral factors, according to a critical review published in September 2025 [122]. While the mechanistic effects of anticholinergic medications on sweating are well established, empirical evidence in this review was limited to low-level sources, such as case reports and series, highlighting a gap in high-quality clinical data. Among patients aged 65 years and older with hospital admissions for acute kidney injury, dehydration, or electrolyte imbalance related to dehydration, approximately 40% of cases were considered possibly preventable if pharmacotherapy of high-risk medications had been timely and adequately adjusted. These medications included diuretics, ACE inhibitors, ARBs, NSAIDs, or metformin [66]. Given the physiologic vulnerability of older adults to renal impairment, it is imperative that clinicians adequately review medications with potential effects on fluid-electrolyte balance and other renal parameters and that ambient temperature be considered, as heat can exacerbate these adverse effects.

Many educational resources and public health efforts exist to mitigate the impact of extreme heat on human health. This scoping review highlights practical resources in the available grey literature that may assist patients, healthcare providers, and those involved in public health planning for older adults. The evaluation literature on these resources highlights effective strategies and factors influencing implementation, including partnerships, leadership support, staffing, and policy, and points to methodological challenges in evaluating the outcomes of such efforts [123,124]. The U.S. CDC Heat & Health Tracker is a notable example of such resources, providing the public with on-demand information about their heat risk based on geographic location and simple risk factors [76]. However, it would be interesting to investigate the demographic data for users of this online tool, since older adults may benefit the most from this resource, but may be less likely to use the Internet than other age groups [125]. Notably, there is a paucity of information for clinicians on the specifics of drug-heat interactions, and available resources have minimal information on medication management strategies for at-risk patients [81]. Twenty-four documents from the U.S. FDALabel database were identified in the search and included in this review. Valuable information is available to prescribers in the “Warnings and Precautions” and “Patient Counseling Information” sections of these documents. Notably, the hyperthermia warning for transdermal scopolamine was added to the medication’s SPL in May 2025, suggesting growing awareness of heat risk among pharmaceutical companies and regulatory agencies such as the U.S. FDA [108].

This scoping review has elucidated numerous gaps in the literature and outstanding questions. Primarily, it is poorly understood how different risk factors for heat-related illness accumulate and potentially synergize. Studies with larger populations should be conducted to evaluate the specific effects of comorbidities, normal age-related physiological changes (i.e., pharmacokinetic changes), gender differences, and polypharmacy on heat susceptibility. Social determinants of health, such as social isolation or access to air conditioning, are also important for a holistic understanding of heat risk to inform targeted efforts to improve health outcomes. The current evidence base for drug-heat interactions is characterized by contradictory results across various drug classes, necessitating more robust, prospective research to validate mechanistic hypotheses. For example, the mechanisms by which certain antihypertensive agents (e.g., diuretics, ACE inhibitors, and ARBs) may increase the risk of heat-related illness should be thoroughly investigated, given their widespread use for hypertension [126]. Additionally, future research should investigate the extent to which a disease itself may increase heat risk, rather than the drug used to treat it. This is relevant to schizophrenia, as discussed above. The exacerbation of hypertension and other cardiovascular conditions leading to excess mortality during extreme heat should also be considered. There are knowledge gaps regarding mitigation strategies as well. Little research has been done to determine how older adults use available information or elucidate best practices for healthcare providers to convey heat-related health risks effectively to patients. Finally, a key outstanding question is what should be done once drug-heat interactions in older adults are better identified. It is not feasible to change the dosing regimen during periods of extreme heat for many drugs and, for many patients, being counseled to avoid outdoor activities is not practical or even possible. There is also a lack of evidence related to which medications or administration strategies may be protective during extreme heat, although early epidemiological data point to possibilities (e.g., empiric potassium use with loop diuretics, statin use in hypercholesterolemia). Medication management during heat waves for at-risk patients will likely require collaboration of a multi-disciplinary team of physicians, pharmacists, and other providers. Integrating heat-related health information into electronic health records could also represent a valuable approach.

Although not specific to older adults and not included in this scoping review, the Americares/Harvard Chan C-CHANGE Climate Resilience for Frontline Clinics Toolkit (2022, updated 2024) provides an additional well-developed resource in the grey literature for extreme heat planning in clinical settings, including information for clinicians, patients, and healthcare administrators. This includes clinical guidance, checklists for clinic staff, and patient-oriented materials to educate and develop Heat Action Plans for those at the highest risk, including older adults and those taking medications that may increase heat sensitivity. This resource was developed by expert working groups and evaluated over four years, including via a national U.S.-based cross-sectional needs assessment, a subgroup analysis of healthcare administrators, and a qualitative post-implementation assessment in 15 pilot clinics. These publications highlight clinic demand for climate resilience resources, the challenges of information overload and patient literacy, and the need for future research on long-term implementation in routine clinical care and its impact on patient outcomes [127,128].

Guidance from major public health bodies and pharmacy organizations outpaces some of the available evidence included in this review. In addition to medication-related risks highlighted by resources such as those from the U.S. CDC and NIA, international policy and educational guidance from the International Pharmaceutical Federation have highlighted the role of pharmacists in educating patients about heat-related risks and in participating in public health planning [129]. Similarly, the American Society of Health-System Pharmacists has highlighted the potential for pharmacists to contribute to climate resilience and protect population health from climate-related hazards, including extreme heat [130,131]. Despite the clear role for pharmacists as accessible health professionals and medication experts, the gap in evidence to guide clinical decision-making and implementation of heat-related medication guidance is a significant limitation. At this time, guidance is primarily limited to medication reviews and patient education on risk, exposure, and self-monitoring. Evidence-based protocols for prescribing decisions are currently lacking, as are key implementation studies to guide integration of heat-related medication considerations into clinical care and population health efforts. These findings point to an opportunity to close the evidence-practice gap through expanded research in this area, including controlled experimental data investigating drug-heat interactions across the lifespan and increasing attention to medication-related adverse effects in epidemiologic studies that incorporate the effects not only of medication class but also of medication dose, medication burden, and within-class effects. Priorities for future research should also include health outcomes data related to dose adjustment during heat events, evaluation of pharmacist-led heat interventions, and implementation research on integrating heat-health workflows for high-risk populations into various pharmacy practice settings.

Several additional limitations were identified in this scoping review. Inherent limitations of scoping review methodology (e.g., the exploratory description of what is known about heat-related illness and medications in this population, inventory and mapping of broad evidence without deep analysis, and incorporation of non-peer-reviewed sources that can reflect potential bias) should be recognized. Direct clinical guidance should not be gleaned from this manuscript. Nearly all of the studies identified through this review were retrospective analyses, and although epidemiological evidence and case reports can be beneficial, it is difficult to establish causality or control external variables when conducting such studies. Synthesizing information is challenging due to the wide variety of study designs, quality, and outcomes of interest. It is also critical to recognize the potential for bias when evaluating the results of an observational study. By definition, scoping reviews do not require critical appraisal of sources [16,17]. This should be kept in mind when reflecting on the present study. Another limitation was the grey literature search, especially the U.S. FDALabel database search and results. Table 2 is unlikely to be a comprehensive list of drugs with heat-related warnings due to the search strategy; for example, if a medication’s SPL was not uploaded in such a way that the database recognized specific subsections, then it may have been left out of the search because it did not contain a “Warnings and Precautions” section. Table 2 should therefore be considered an educational sample of drug-heat regulatory information rather than a comprehensive list. It is also important to note that the definitions of a diagnosis of heat-related illness used for this review may not fully capture the range of adverse health outcomes known to be associated with exposure to extreme heat, such as cardiovascular, respiratory, or endocrine and metabolic morbidity and mortality [132]. Nonetheless, this is the first scoping review to examine heat sensitivity and medication risk in older adults, a particularly significant finding given the rapidly aging populations of many countries [133]. Our comprehensive search strategy enabled thorough mapping of the primary literature to identify commonalities and research gaps.

## 5. Conclusions

The current body of literature addressing the role of medications in heat-related illness risk in older adults remains heterogeneous in design and often inconsistent in results. Much of the cited risk relies on physiological plausibility and epidemiological data rather than controlled experimental data. This scoping review highlights the need for more studies investigating the intersection of age, multimorbidity, medication use, and heat-related illness to inform future mitigation efforts. As heat events become more frequent, intense, and widespread, interactions among medications, age, and extreme heat will become a key dimension of medication safety for older adults. Pharmacists are well-positioned to make significant contributions to clinical, research, and public health responses to this emerging challenge. Closing the gap between available evidence and translation to practice will be an essential step to ensure the success of efforts to protect the health of older adults in a changing climate.

## Figures and Tables

**Table 1:** Drug Classes with the Highest Risk of Heat-Related Health Effects.

Therapeutic Effect	Drug (s)	Results	Study Type	Study Author & Year
Cardiovascular	Hyperthermia-Related Conditions			
	Diuretics	Furosemide was among the most common drugs in patients with primary hyperthermia	Cohort	Bongers 2020 [22]
	Diuretics	No association between heat-related GP consultations and diuretics in patients with diabetes	Case-crossover	Hajat 2017 [59]
	Diuretics	Chronic treatment with diuretics was one independent prognostic factor found in non-exertional heatstroke	Cohort	Hausfater 2010 [23]
	Diuretics	Diuretic use was associated with mortality among heatstroke ICU patients in univariate analysis, but not in multivariate analysis	Retrospective risk-factor	Misset 2006 [33]
	Diuretics, cardiotropes	No significant association between heat-related death and diuretics or cardiotropes in ED patients	Retrospective observational	Davido 2006 [19]
	Anticoagulants, nitrates, diuretics, beta blockers, calcium channel blockers, ACE inhibitors, ARBs	Increased relative risk of hospital admission for heat-related illness or dehydration upon initiation of anticoagulants, nitrates, diuretics, beta blockers, calcium channel blockers, ACEI, and ARBs; risk was highest for ACEI in combination with a diuretic	Sequence symmetry analysis	Kalisch 2016 [36]
	ACE inhibitors, ARBs, loop diuretics	ACE inhibitors, ARBs, and loop diuretics were associated with increased risk of heat-related hospitalization in the summer months among older Medicare beneficiaries with chronic conditions; no significant synergistic effects were found	Case series	Layton 2020 [28]
	Antihypertensives, general	Syncope cases were more frequent in the summer months among those taking antihypertensives; antihypertensive medications may excessively lower blood pressure in hot and dry climates, leading to more frequent syncopal episodes and dehydration	Case-control	Huang 2014 [29]
	Antihypertensives, general	Syncope cases were more frequent in the summer months among those taking antihypertensive medications, but no specific drug class had a greater association; significant increase in cases of syncope secondary to dehydration during the summer months (40.5%) vs. winter months (29%) in patients taking antihypertensives	Case-control extension of [29]	Huang 2015 [30]
	Antihypertensives, general	Antihypertensive medication did not affect critical environmental limits among study subjects in warm-humid or hot-dry conditions	Randomized, double-blind, placebo-controlled crossover	Leach 2025 [64]
	Aspirin	Low-dose aspirin did not alter core or skin temperatures among study subjects in warm-humid or hot-dry conditions; it did reduce skin blood flow responses to heat stress	Randomized, double-blind, placebo-controlled crossover	Fisher 2025 [65]
	Dehydration or Electrolyte Imbalances			
	Thiazide diuretics, ACE inhibitors, ARBs	Thiazide diuretics, ACE inhibitors, and ARBs were more associated with biochemical parameters of impaired renal function in the summer than in the winter in elderly patients	Cohort	Barski 2015 [43]
	Diuretics, ACE inhibitors, ARBs	40% of acute kidney injury (AKI) and dehydration admissions in elderly patients were considered possibly preventable if pharmacotherapy of high-risk medications had been timely and adequately adjusted (including diuretics, ACE inhibitors, and ARBs)	Case series	Coppes 2024 [66]
	Diuretics, ACE inhibitors, ARBs	ADRs reported during summer 2003 in France were most commonly metabolic or neuropsychiatric; diuretics, ACE inhibitors, and ARBs were among the most common drugs	Retrospective observational	Michenot 2006 [46]
	Hydrochlorothiazide, plus other cardiovascular drugs (and nervous system drugs)	Significant association between drug-induced severe hyponatremia and high environmental temperature during 10-year pharmacovigilance program; hydrochlorothiazide, other cardiovascular drugs, and nervous system drugs were the most frequent culprits	Prospective evaluation	Ramírez 2019 [45]
	Thiazide diuretics, ACE inhibitors, ARBs	Higher daily temperature was associated with impaired renal function in older adults treated with thiazides, ACE inhibitors, or ARBs	Cohort	Sagy 2016 [67]
	Diuretics	Significant increase in diuretic-induced hyponatremia with increase in temperature (4% per degree Celsius) in ED patients	Cross-sectional cohort	Sailer 2019 [40]
	Diuretics	No statistically significant association between diuretics and profound hyponatremia in warmer months among patients admitted for profound hyponatremia	Cross-sectional	Sasaki 2019 [39]
	Beta blockers, ACE inhibitors, ARBs (and insulin)	No significant association between hyperkalemia and beta blockers, ACE inhibitors, or ARBs (or insulin) among hemodialysis patients	Retrospective observational	Tsigaka 2022 [42]
	General or Unspecified Outcome			
	Cardiovascular drugs, general	Use of cardiovascular medication was the most prevalent risk factor found among healthy older adults in Quebec	Cross-sectional	Laverdière 2015 [62]
	Diuretics, ACE inhibitors	Significantly more ADRs were heat-related in 2003 and 2006 (summers with heat waves) than in the reference period; diuretics and ACE inhibitors were among the most frequently involved drugs	Retrospective observational	Sommet 2012 [47]
Psychotropic	Hyperthermia-Related Conditions			
	Antipsychotics	Antipsychotics were associated with increased risk of heat-related hospitalization in the summer months among older Medicare beneficiaries with chronic conditions; no significant synergistic effects were found	Case series	Layton 2020 [28]
	Antipsychotics, anxiolytics	Antipsychotics and anxiolytics were associated with ED admission for heatstroke or hyperthermia	Matched case-control	Martin-Latry 2007 [31]
	Antipsychotics, antidepressants	No association between heat-related GP consultations and antipsychotics or antidepressants in patients with diabetes	Case-crossover	Hajat 2017 [59]
	Antipsychotics, antidepressants, anxiolytics	Increased relative risk of hospital admission for heat-related illness or dehydration upon initiation of antipsychotics, antidepressants, and anxiolytics	Sequence symmetry analysis	Kalisch 2016 [36]
	Psychotropics, general	Survivors were less often on psychotropic medications than non-survivors in ED patients evaluated for heat-related pathologies	Retrospective observational	Davido 2006 [19]
	Psychotropics, general	No significant association between psychotropic medication use and heat-related illness among people with mental illness; 40–60% increased risk for this population regardless of medications	Self-controlled case series	Wong 2024 [68]
	Dehydration or Electrolyte Imbalances			
	Antidepressants	ADRs reported during summer 2003 in France were most commonly metabolic or neuropsychiatric; antidepressants were among the most common drugs	Retrospective observational	Michenot 2006 [46]
	Lithium	No significant association between renal parameters or other laboratory tests with environmental temperature in lithium users	Cross-sectional	Rej 2014 [69]
	General or Unspecified Outcome			
	Antidepressants, antipsychotics, benzodiazepines, non-benzodiazepine anxiolytics/hypnotics	Significant dose-response relationship between the number of psychotropic drugs and risk of mortality in older adults; antidepressants, Selective Serotonin Reuptake Inhibitors (SSRIs) only, and antipsychotics (“other” only) were associated with a 20% and 40% increased risk of death before heatwave; antidepressants and antipsychotics (all) were associated with a 70% and 110% increased risk of death during heat wave; anxiolytics/hypnotics were associated with a decreased risk before heatwave, but during heatwave, benzodiazepines had no association and non-benzodiazepine anxiolytics/hypnotics were associated with increased risk	Case-control	Nordon 2009 [70]
	Antidepressants	Significantly more ADRs were heat-related in 2003 and 2006 (summers with heat waves) than in the reference period; serotonin antidepressants were among the most frequently involved drugs	Retrospective observational	Sommet 2012 [47]
Anticholinergic	Hyperthermia-Related Conditions			
	Benzatropine	Use of benzatropine (plus amisulpride, amlodipine + olmesartan, or aclidinium) may have impaired thermoregulation of patient with multiple comorbidities experiencing homelessness, leading to heatstroke	Case series	English 2022 [71]
	Oxybutynin	Use of oxybutynin may have exacerbated effects of extreme heat and strenuous exercise in elderly patient, leading to fatal heatstroke	Case report	Herbst 2011 [24]
	Anticholinergics, general	No association between heat-related GP consultations and anticholinergics in patients with diabetes	Case-crossover	Hajat 2017 [59]
	Anticholinergics, general	Increased relative risk of hospital admission for heat-related illness or dehydration upon initiation of anticholinergics	Sequence symmetry analysis	Kalisch 2016 [36]
	Anticholinergics, general	Anticholinergics were associated with increased risk of heat-related hospitalization in the summer months among older Medicare beneficiaries with chronic conditions; no significant synergistic effects were found	Case series	Layton 2020 [28]
	Anticholinergics, general	Anticholinergics were associated with ED admission for heatstroke or hyperthermia	Matched case-control	Martin-Latry 2007 [31]
Other	Hyperthermia-Related Conditions			
	Antiepileptics, levothyroxine	Antiepileptics were among the most common drugs in patients with primary hyperthermia; high rate of levothyroxine use in study population compared to national average	Cohort	Bongers 2020 [22]
	Antiepileptics, NSAIDs, anti-Parkinson’s agents, hypnotics, antihistamines	Increased relative risk of hospital admission for heat-related illness or dehydration upon initiation of NSAIDs; no significant association for antiepileptics, anti-Parkinson’s agents, hypnotics, and antihistamines	Sequence symmetry analysis	Kalisch 2016 [36]
	Dehydration or Electrolyte Imbalances			
	NSAIDs, metformin	40% of AKI and dehydration admissions in elderly patients were considered possibly preventable if pharmacotherapy of high-risk medications had been timely and adequately adjusted (including NSAIDs and metformin)	Case series	Coppes 2024 [66]
	Sodium-glucose co-transporter 2 (SGLT2) inhibitors	Higher incidence of dehydration-related ADRs during the summer months	Retrospective observational	Matsumoto 2024 [72]
	General or Unspecified Outcome			
	Proton pump inhibitors (PPIs)	Significantly more ADRs were heat-related in 2003 and 2006 (summers with heat waves) than in the reference period; among the most frequently involved medications	Retrospective observational	Sommet 2012 [47]
Not Specified		Hyperthermia-Related Conditions		
		Among hospital admissions for the effects of heat and light, 2.4% of external causes were “drug-related”	Retrospective observational	Beggs 2008 [18]
		Dehydration or Electrolyte Imbalances		
		Odds ratio for drug-induced hyponatremia increased with increasing temperature during summer months among ADR reports; the change in sodium per 1 degree Celsius was estimated to be −0.37 mmol/L	Case-crossover	Jönsson 2017 [44]

**Table 2:** U.S. FDA SPL for Identified Medications with Heat-Related Warnings.

Generic Drug Name (Class)	Warnings and Precautions	Patient Counseling Information
Aripiprazole tablet (atypical antipsychotic) [89]	Body Temperature Regulation: Disruption of the body’s ability to reduce core body temperature has been attributed to antipsychotic agents. Appropriate care is advised when prescribing aripiprazole for patients who will be experiencing conditions that may contribute to an elevation in core body temperature (e.g., exercising strenuously, exposure to extreme heat, receiving concomitant medication with anticholinergic activity, or being subject to dehydration).	Heat Exposure and Dehydration: Patients should be advised regarding appropriate care in avoiding overheating and dehydration.
Asenapine transdermal film (atypical antipsychotic) [90]	Body Temperature Regulation: Atypical antipsychotics may disrupt the body’s ability to reduce core body temperature. Strenuous exercise, exposure to extreme heat, dehydration, and anticholinergic medications may contribute to an elevation in core body temperature; use asenapine with caution in patients who may experience these conditions.	Heat Exposure and Dehydration: Counsel patients regarding appropriate care in avoiding overheating and dehydration.
Brexipiprazole tablet (atypical antipsychotic) [91]	Body Temperature Dysregulation: Atypical antipsychotics may disrupt the body’s ability to reduce core body temperature. Strenuous exercise, exposure to extreme heat, dehydration, and anticholinergic medications may contribute to an elevation in core body temperature; use brexipiprazole with caution in patients who may experience these conditions.	Heat Exposure and Dehydration: Counsel patients regarding appropriate care in avoiding overheating and dehydration.
Cariprazine capsule (atypical antipsychotic) [92]	Body Temperature Dysregulation: Atypical antipsychotics may disrupt the body’s ability to reduce core body temperature. Strenuous exercise, exposure to extreme heat, dehydration, and anticholinergic medications may contribute to an elevation in core body temperature; use cariprazine with caution in patients who may experience these conditions.	Heat Exposure and Dehydration: Educate patients regarding appropriate care in avoiding overheating and dehydration.
Dicyclomine hydrochloride tablet (anticholinergic) [93]	Peripheral and Central Nervous System: […] In the presence of high environmental temperature, heat prostration can occur with drug use (fever and heat stroke due to decreased sweating).	Peripheral and Central Nervous system: […] In the presence of a high environmental temperature, heat prostration can occur with dicyclomine hydrochloride use (fever and heat stroke due to decreased sweating). If symptoms occur, the drug should be discontinued and a physician contacted.
Empagliflozin film-coated tablet (SGLT2 inhibitor) [94]	AKI and Impairment in Renal Function: […] Consider temporarily discontinuing empagliflozin in any setting of reduced oral intake (such as acute illness or fasting) or fluid losses (such as gastrointestinal illness or excessive heat exposure); monitor patients for signs and symptoms of acute kidney injury. If acute kidney injury occurs, discontinue empagliflozin promptly and institute treatment.	AKI: Inform patients that acute kidney injury has been reported during use of empagliflozin. Advise patients to seek medical advice immediately if they have reduced oral intake (such as due to acute illness or fasting) or increased fluid losses (such as due to vomiting, diarrhea, or excessive heat exposure), as it may be appropriate to temporarily discontinue empagliflozin use in those settings.
Glycopyrrolate tablet (anticholinergic) [95]	Heat Prostration at High Environmental Temperatures: In the presence of a high environmental temperature, heat prostration resulting in fever and heatstroke can occur with the use of glycopyrrolate tablets due to decreased sweating, particularly in geriatric patients [see Adverse Reactions (6)]. Advise patients to avoid exposure to hot or very warm environmental temperatures when taking glycopyrrolate tablets. Glycopyrrolate tablets are not recommended in geriatric patients.	Heat Prostration at High Environmental Temperatures: Inform patients that Glycopyrrolate tablets can reduce sweating, leading to the possibility of heat exhaustion or heat stroke. Advise patients to avoid exposure to hot or very warm environmental temperatures.
	Increased Risk of Anticholinergic Adverse Reactions in Geriatric Patients: Geriatric patients 65 years of age and older are at increased risk of anticholinergic adverse reactions that may lead to complications of urinary retention, bowel obstruction, heat prostration, arrhythmias, delirium, and falls or fractures. Glycopyrrolate tablet 1 mg and glycopyrrolate tablet 2 mg are not recommended in geriatric patients and may be contraindicated in some geriatric patients with underlying medical conditions.	
Glycopyrronium cloth (anticholinergic) [96]	Control of Body Temperature: In the presence of high ambient temperature, heat illness (hyperpyrexia and heat stroke due to decreased sweating) can occur with the use of anticholinergic drugs such as glycopyrronium. Advise patients using glycopyrronium to watch for generalized lack of sweating when in hot or very warm environmental temperatures and to avoid use if not sweating under these conditions.	Control of Body Temperature (Risk of Overheating or Heat Illness): In the presence of high ambient temperature, heat illness due to decreased sweating can occur with the use of anticholinergic drugs such as glycopyrronium. Advise patients using glycopyrronium to watch for generalized lack of sweating when in hot or very warm environmental temperatures and to avoid use if not sweating under these conditions.
Iloperidone tablet (atypical antipsychotic) [97]	Body Temperature Regulation: Atypical antipsychotics may disrupt the body’s ability to reduce core body temperature. Strenuous exercise, exposure to extreme heat, dehydration, and anticholinergic medications may contribute to an elevation in core body temperature; use iloperidone with caution in patients who may experience these conditions.	Heat Exposure and Dehydration: Educate patients regarding appropriate care in avoiding overheating and dehydration
Lumateperone capsule (atypical antipsychotic) [98]	Body Temperature Dysregulation: Atypical antipsychotics may disrupt the body’s ability to reduce core body temperature. Strenuous exercise, exposure to extreme heat, dehydration, and anticholinergic medications may contribute to an elevation in core body temperature; use lumateperone with caution in patients who may experience these conditions.	Heat Exposure and Dehydration: Educate patients regarding appropriate care in avoiding overheating and dehydration.
Lurasidone HCl tablet (atypical antipsychotic) [99]	Body Temperature Dysregulation: Disruption of the body’s ability to reduce core body temperature has been attributed to antipsychotic agents. Appropriate care is advised when prescribing lurasidone hydrochloride for patients who will be experiencing conditions that may contribute to an elevation in core body temperature, e.g., exercising strenuously, exposure to extreme heat, receiving concomitant medication with anticholinergic activity, or being subject to dehydration.	Heat Exposure and Dehydration: Educate patients regarding appropriate care in avoiding overheating and dehydration.
Olanzapine tablet (atypical antipsychotic) [100]	Body Temperature Regulation: Disruption of the body’s ability to reduce core body temperature has been attributed to antipsychotic agents. Appropriate care is advised when prescribing olanzapine for patients who will be experiencing conditions that may contribute to an elevation in core body temperature, e.g., exercising strenuously, exposure to extreme heat, receiving concomitant medication with anticholinergic activity, or being subject to dehydration.	Body Temperature Regulation: Patients should be advised regarding appropriate care in avoiding overheating and dehydration. Patients should be advised to call their doctor right away if they become severely ill and have some or all of these symptoms of dehydration: sweating too much or not at all, dry mouth, feeling very hot, feeling thirsty, not able to produce urine.
Olanzapine and fluoxetine capsule (atypical antipsychotic and SSRI) [101]	Body Temperature Regulation: Disruption of the body’s ability to reduce core body temperature has been attributed to antipsychotic agents. Appropriate care is advised when prescribing olanzapine and fluoxetine capsules for patients who will be experiencing conditions that may contribute to an elevation in core body temperature, e.g., exercising strenuously, exposure to extreme heat, receiving concomitant medication with anticholinergic activity, or being subject to dehydration.	Body Temperature Regulation: Patients should be advised regarding appropriate care in avoiding overheating and dehydration. Patients should be advised to call their doctor right away if they become severely ill and have some or all of these symptoms of dehydration: sweating too much or not at all, dry mouth, feeling very hot, feeling thirsty, not able to produce urine.
Olanzapine and samidorphan L-malate tablet (atypical antipsychotic and opioid antagonist) [102]	Body Temperature Dysregulation: Atypical antipsychotics may disrupt the body’s ability to reduce core body temperature. Strenuous exercise, exposure to extreme heat, dehydration, and anticholinergic medications may contribute to an elevation in core body temperature; use olanzapine/samidorphan with caution in patients who may experience these conditions.	Body Temperature Dysregulation: Educate patients regarding appropriate care in avoiding overheating and dehydration.
Paliperidone tablet (atypical antipsychotic) [103]	Body Temperature Dysregulation: Disruption of the body’s ability to reduce core body temperature has been attributed to antipsychotic agents. Appropriate care is advised when prescribing paliperidone to patients who will be experiencing conditions that may contribute to an elevation in core body temperature, e.g., exercising strenuously, exposure to extreme heat, receiving concomitant medication with anticholinergic activity, or being subject to dehydration.	Heat Exposure and Dehydration: Counsel patients on the importance of avoiding overheating and dehydration.
Phentermine and topiramate capsule (anorexiant) [104]	Oligohidrosis and Hyperthermia: Oligohidrosis (decreased sweating), infrequently resulting in hospitalization, has been reported in association with the use of topiramate. Decreased sweating and an elevation in body temperature above normal characterized these cases. Some of the cases have been reported with topiramate after exposure to elevated environmental temperatures.	Oligohidrosis and Hyperthermia: Inform patients that oligohidrosis (decreased sweating) has been reported in association with the use of topiramate, particularly in pediatric patients. Advise patients to monitor for decreased sweating and increased body temperature during physical activity, especially in hot weather.
	The majority of the reports associated with topiramate have been in pediatric patients. Advise all patients and caregivers to monitor for decreased sweating and increased body temperature during physical activity, especially in hot weather. Patients on concomitant medications that predispose them to heat-related disorders may be at increased risk.	
Prochlorperazine maleate tablet (anti-emetic/ antipsychotic) [105]	Because phenothiazines may interfere with thermoregulatory mechanisms, use with caution in persons who will be exposed to extreme heat.	N/A
Quetapine tablet (atypical antipsychotic) [106]	Body Temperature Regulation: Disruption of the body’s ability to reduce core body temperature has been attributed to antipsychotic agents. Appropriate care is advised when prescribing quetiapine for patients who will be experiencing conditions that may contribute to an elevation in core body temperature, e.g., exercising strenuously, exposure to extreme heat, receiving concomitant medication with anticholinergic activity, or being subject to dehydration.	Heat Exposure and Dehydration: Patients should be advised regarding appropriate care in avoiding overheating and dehydration
Risperidone subcutaneous (atypical antipsychotic) [107]	Body Temperature Regulation: Atypical antipsychotics may disrupt the body’s ability to reduce core body temperature. Both hyperthermia and hypothermia have been reported in association with oral risperidone use. Strenuous exercise, exposure to extreme heat, dehydration, and anticholinergic medications may contribute to an elevation in core body temperature; use risperidone with caution in patients who may experience these conditions.	Heat Exposure and Dehydration: Educate patients regarding appropriate care in avoiding overheating and dehydration.
Scopolamine patch (anticholinergic) [108]	Hyperthermia: Serious adverse reactions of hyperthermia have been reported postmarketing in adult and pediatric patients receiving transdermal scopolamine, including fatal cases. Anticholinergic agents, including scopolamine, can increase core body temperature and reduce sweating, which may cause further increases in body temperature. Hyperthermia may be exacerbated by exposure to external heat sources or high environmental temperature. Pediatric and geriatric patients may be more susceptible to these anticholinergic effects on thermoregulation. Advise patients if body temperature increases or they are not sweating in warm environmental conditions to remove the transdermal system and contact their healthcare provider. Symptoms may persist following removal of the used transdermal system as there may be continued systemic absorption of scopolamine through the skin. Scopolamine transdermal system is not approved for use in pediatric patients.	Hyperthermia: Inform patients that scopolamine transdermal system can increase body temperature and reduce sweating, which may result in hyperthermia and be exacerbated by exposure to external heat sources or high environmental temperature. Geriatric patients may be more susceptible to these effects. Advise patients if body temperature increases or if they are not sweating in warm environmental conditions to remove the transdermal system and contact their healthcare provider. Symptoms may persist after removal of the transdermal system.
Sofironium bromide gel (anticholinergic) [109]	Control of Body Temperature: In the presence of high ambient temperature, heat illness (hyperpyrexia and heat stroke due to decreased sweating) can occur with the use of anticholinergic drugs, including sofironium. Watch for generalized lack of sweating when in hot or very warm environmental temperatures and avoid using sofironium if not sweating under these conditions.	Control of Body Temperature: Advise patients that in the presence of high ambient temperature, heat illness due to decreased sweating can occur with the use of sofironium. Advise patients to watch for generalized lack of sweating when in hot or very warm environmental temperatures and avoid using sofironium if not sweating under these conditions.
Topiramate tablet (anticonvulsant) [110]	Oligohidrosis and Hyperthermia: Oligohidrosis (decreased sweating), infrequently resulting in hospitalization, has been reported in association with the use of topiramate. Decreased sweating and an elevation in body temperature above normal characterized these cases. Some of the cases have been reported with topiramate after exposure to elevated environmental temperatures.	Oligohidrosis and Hyperthermia: Closely monitor topiramate-treated patients, especially pediatric patients, for evidence of decreased sweating and increased body temperature, especially in hot weather. Counsel patients to contact their healthcare professionals immediately if they develop a high or persistent fever or show decreased sweating.
	The majority of the reports associated with topiramate have been in pediatric patients. Advise all patients and caregivers to monitor for decreased sweating and increased body temperature during physical activity, especially in hot weather. Patients on concomitant medications that predispose them to heat-related disorders may be at increased risk.	
Ziprasidone capsule (atypical antipsychotic) [111]	Body Temperature Regulation: Although not reported with ziprasidone in premarketing trials, disruption of the body’s ability to reduce core body temperature has been attributed to antipsychotic agents. Appropriate care is advised when prescribing ziprasidone for patients who will be experiencing conditions that may contribute to an elevation in core body temperature, e.g., exercising strenuously, exposure to extreme heat, receiving concomitant medication with anticholinergic activity, or being subject to dehydration.	N/A
Zonisamide suspension (anticonvulsant) [112]	Oligohidrosis and Hyperthermia in Pediatric Patients: […] Pediatric patients appear to be at an increased risk for zonisamide-associated oligohidrosis and hyperthermia. Patients, especially pediatric patients, treated with zonisamide should be monitored closely for evidence of decreased sweating and increased body temperature, especially in warm or hot weather. Caution should be used when zonisamide is prescribed with other drugs that predispose patients to heat-related disorders; these drugs include, but are not limited to, carbonic anhydrase inhibitors and drugs with anticholinergic activity.	Oligohidrosis and Hyperthermia in Pediatric Patients: Patients should contact their physician immediately if a child has been taking zonisamide and is not sweating as usual, with or without a fever.

## Data Availability

No new data were created or analyzed in this study. Data sharing is not applicable to this article.

## Supplementary Materials

The following supporting information can be downloaded at https://www.mdpi.com/article/10.3390/pharmacy14030074/s1, File S1: Literature Search Strategy; Table S1: Data Extraction Tool; Table S2: Sources of Evidence; Table S3: Scientific Literature Characteristics; Table S4: Grey Literature Characteristics.
