# Supplementary material for: Recognizing and Mitigating the Effects of Medication on Heat-Related Illness in Older Adults: A Scoping Review
Source: Pharmacy (Basel). 2026 May 17;14(3):74. doi: 10.3390/pharmacy14030074 (PMC13214780; doi:10.3390/pharmacy14030074)
Supplement: Supplementary file 1 [file pharmacy-14-00074-s001.zip › pharmacy-4202586-Revision 2 SM.pdf]

pharmacy

File S1. Literature Search Strategy

Embase:

('heat injury'/exp OR 'heat injury' OR 'heat stroke' OR 'heat exhaustion' OR 'heat syncope' OR 'dehydration'/exp OR 'dehydration' OR 'electrolyte disturbance'/exp OR 'electrolyte imbalance' OR 'hyponatremia' OR 'hypernatremia' OR 'hyperkalemia' OR 'hypokalemia' OR 'thermoregulation'/exp OR 'thermoregulation' OR 'hyperthermia' OR 'heat sensitivity'/exp OR 'heat sensitivity' OR 'heat sensitive' OR 'heat intolerance'/exp OR 'heat intolerance') AND ('aged'/exp OR 'aged' OR 'older adult' OR 'elderly' OR 'geriatric disorder'/exp) AND ('heat stress' OR 'heat stress'/exp OR 'heat wave' OR 'heat wave'/exp OR 'summer' OR 'seasonal' OR 'season' OR 'high temperatures' OR 'environmental temperature') AND ('drug therapy'/exp OR 'drug therapy' OR 'medication use' OR 'drug induced disease'/exp OR 'drug associated' OR 'medication associated' OR 'drug interaction' OR 'drug interaction'/exp OR 'adverse drug reaction' OR 'adverse drug reaction'/exp OR 'prescription'/exp OR 'prescription' OR 'antihypertensive agent'/exp OR 'neuroleptic agent'/exp OR 'psychotropic agent'/exp OR 'antidepressant agent'/exp OR 'anticonvulsive agent'/exp OR 'antihistaminic agent'/exp OR 'cholinergic receptor blocking agent'/exp) AND [2000-2025]/py

PubMed:

((((((((((((((("medication use"[Title/Abstract] OR "prescription medication"[Title/Abstract] OR "prescription drug"[Title/Abstract] OR "medication associated"[Title/Abstract] OR "drug associated"[Title/Abstract] OR "medication induced"[Title/Abstract] OR "drug induced"[Title/Abstract] OR "adverse drug reaction"[Title/Abstract] OR "drug interaction"[Title/Abstract]) OR (adverse drug reaction[MeSH Terms])) OR (drug related side effects and adverse reaction[MeSH Terms])) OR (body temperature regulation/drug effects[MeSH Terms])) OR (agent, antihypertensive[MeSH Terms])) OR (agent, antipsychotic[MeSH Terms])) OR (drug, psychotropic[MeSH Terms])) OR (antidepressant[MeSH Terms])) OR (agent, anticholinergic[MeSH Terms])) OR (agent, anticonvulsive[MeSH Terms])) OR (antihistamine[MeSH Terms])) OR (drug[Title/Abstract])) OR (medication[Title/Abstract]) AND (2000:2025[pdat])) (((((((("heat"[Title/Abstract] OR "heat wave"[Title/Abstract] OR "summer"[Title/Abstract] OR "temperature"[Title/Abstract] OR "extreme heat"[Title/Abstract] OR "weather"[Title/Abstract]) OR (heat stress[MeSH Terms])) OR (heat wave[MeSH Terms])) OR (hot weather, extreme[MeSH Terms])) OR (warm[Title/Abstract])) OR (hot[Title/Abstract])) OR ("high temperature"[Title/Abstract])) OR ("environmental temperature"[Title/Abstract])) OR ("ambient temperature"[Title/Abstract]) AND (((("older adult"[Title/Abstract] OR "elderly"[Title/Abstract] OR "aged"[Title/Abstract] OR "senior"[Title/Abstract] OR "elder"[Title/Abstract]) OR (aged[MeSH Terms])) OR ("age"[Title/Abstract])))) AND (((("heat illness"[Title/Abstract] OR "heat-related illness"[Title/Abstract] OR "heat injury"[Title/Abstract] OR "dehydration"[Title/Abstract] OR "heat stroke"[Title/Abstract] OR "heat exhaustion"[Title/Abstract] OR "heat syncope"[Title/Abstract] OR "sunstroke"[Title/Abstract] OR "electrolyte imbalance"[Title/Abstract] OR "hyponatremia"[Title/Abstract] OR "hypokalemia"[Title/Abstract] OR "hyperkalemia"[Title/Abstract] OR "hypernatremia"[Title/Abstract] OR "heat sensitivity"[Title/Abstract] OR "heat intolerance"[Title/Abstract]) OR "heat sensitive"[Title/Abstract] OR (heat stress disorder[MeSH Terms])) OR (balance, water electrolyte[MeSH Terms])) OR (hyperthermia[Title/Abstract])) AND (2000:2025[pdat])

Scopus:

(TITLE-ABS-KEY ("older adult" OR "elderly" OR "aged" OR "senior" OR “elder”) AND TITLE-ABS-KEY ("heat stress" OR "extreme heat" OR "heat wave" OR "warm weather" OR "summer" OR "high temperature" OR "environmental temperature" OR "ambient temperature" OR “seasonal” OR “season”) AND TITLE-ABS-KEY ("medication use" OR "drug therapy" OR "drug associated" OR "medication associated" OR "drug induced" OR "medication induced" OR "adverse drug reaction" OR "drug interaction" OR “antihypertensive” OR “antipsychotic” OR “antidepressant” OR “anticholinergic” OR “antihistamine” OR “anticonvulsive”) AND TITLE-ABS-KEY ("heat injury" OR "heat illness" OR "heat-related illness" OR "heat stroke" OR "heat exhaustion" OR "heat syncope" OR "hyperthermia" OR "dehydration" OR "electrolyte imbalance" OR "electrolyte disturbance" OR "hyponatremia" OR "hypernatremia" OR "hypokalemia" OR "hyperkalemia" OR "thermoregulation" OR "heat sensitivity" OR "heat intolerance" OR “heat sensitive”) AND PUBYEAR > 1999 AND PUBYEAR < 2025

Web of Science:
(((ALL=("elderly" OR "older adult" OR "aged" OR "senior" OR "age" OR “elder”)) AND ALL=("summer" OR "weather" OR "heat wave" OR "extreme heat" OR "high temperature" OR "season” OR “seasonal” OR “temperature”)) AND ALL=("heat stroke" OR "heat related illness" OR "heat illness" OR "heat injury" OR "heat exhaustion" OR "hyperthermia" OR "dehydration" OR "electrolyte imbalance" OR "electrolyte disturbance" OR "hyponatremia" OR "hypernatremia" OR "hypokalemia" OR "hyperkalemia" OR "heat sensitivity" OR "heat intolerance" OR "thermoregulation" OR "thermoregulate" OR "heat sensitive”)) AND ALL=("medication use" OR "prescription” OR "drug associated" OR "medication associated" OR "adverse drug reaction" OR "drug interaction" OR "side effect" OR "prescription" OR "antihypertensive" OR "antipsychotic" OR "psychotropic" OR "anticholinergic" OR "antihistamine" OR “anticonvulsive” OR “beta blocker” OR “diuretic” OR “neuroleptic”) Timespan: 2000-01-01 to 2025-06-01

Table S1. Data Extraction Tool

Basic information	Title	
	Author(s)	
	Year	
	Location	
	Type of evidence source	
For primary studies	Aim	
	Population	
	Methodology	
	Results	
	Heat sensitivity in older adults	

Relevance to scoping review	Medication use	
	Guidance or recommendations to mitigate impact	
For grey literature	Intended audience	
	Origin	
	Format	
	Information provided	

Table S2. Sources of Evidence

Basic Information		*Rationale for Inclusion*		
**Title, Author, and Year**	**Type of Study or Source**	**Heat Sensitivity in Older Adults**	**Medication Use**	**Guidance or Strategies to Mitigate Impact**
Primary Literature				
Identifying Risk Factors for Hospitalization with Behavioral Health Disorders and Concurrent Temperature-Related Illness in New York State (2022) Aydin-Ghormoz H; Adeyeye T; Muscatiello N; Nayak S; Savadatti S; Insaf TZ	Cross-sectional study	Increasing age, dementia (RR 1.65; 95% CI: 1.49, 1.83), and schizophrenia (RR 1.38 95% CI: 1.19, 1.60) were among the risk factors for concurrent behavioral health disorders and heat-related illness	Briefly discusses thermoregulatory disturbance due to antipsychotic medication use	N/A
Seasonal influence on the renal function in hospitalized elderly patients (2015) Barski, L.; Bartal, C.; Sagy, I.; Jotkowitz, A.; Nevzorov, R.; Zeller, L.; Dizengof, V.; Rogachev, B.	Cohort study	Extensive fluid loss in the summer may lead to mild renal impairment in a population of hospitalized older adults, but this may not be clinically significant due to physiological mechanisms	Thiazide diuretics, ACE-inhibitors, and ARBs were more associated with the biochemical parameters of impaired renal function in the summer than in the winter	N/A
Admission to hospital for effects of heat and light: NSW, 1993-94 to 2003-04 (2008) Beggs, P.J.; Vaneckova, P.	Retrospective observational study	39% of admissions with the effects of heat and light as principal diagnosis were aged 65 years and older	A small percentage (2.4%) of external causes were drug-related	N/A
Drug-associated hyperthermia: A longitudinal analysis of hospital presentations (2020) Bongers KS; Salahudeen MS; Peterson GM	Cohort study	116 patients (51.8%) presenting with hyperthermia during the study period were 60+ years old; higher mean Charlson Comorbidity Index (CCI) and number of drugs in older group	Possible association of diuretics, antiepileptics, and levothyroxine with hyperthermia (furosemide and antiepileptics were the most common drugs in patients with primary hyperthermia and levothyroxine use in study population was high compared to national average)	N/A
Impacts of extreme heat on emergency medical service calls in King County, Washington, 2007-2012: relative risk and time series analyses of basic and advanced life support. (2016)	Cohort study	Notably, the 65-84 and 85+ age groups were NOT associated with the greatest risk in this study	N/A	N/A

Calkins MM; Isaksen TB; Stubbs BA; Yost MG; Fenske RA				
Help-seeking behavior during elevated temperature in Chinese population (2011) Chan, E.Y.; Goggins, W.B.; Kim, J.J.; Griffiths, S.; Ma, T.K.	Time-series study	Individuals 75+ made up the greatest proportion of health-related emergency calls, had lower thresholds at which high temperatures affected emergency calls, and were more sensitive to higher maximum temperatures	N/A	N/A
Influence of climate on the incidence of thiazide-induced hyponatraemia (2007) Chow, K.M.; Szeto, C.C.; Kwan, B.C.-H.; Li, P.K.-T	Cohort study	Mean age of patients diagnosed with thiazide-induced hyponatremia was 76 ±6	Investigators did NOT find an association between thiazide-induced hyponatremia and temperature	N/A
Heat-related fatalities in Wisconsin during the summer of 2012 (2013) Christenson ML; Geiger SD; Anderson HA	Case series	Age, cardiovascular disease, and mental illness were identified as risk factors; diminished ability to thermoregulate is discussed	Risk of antipsychotic medication is briefly discussed	N/A
Characteristics and preventability of medication-related admissions for acute kidney injury and dehydration in elderly patients (2024) Coppes T; Hazen ACM; Zwart DLM; Koster ES; van Gelder T; Bouvy ML	Case series	Cases were patients aged ≥65 years admitted for medication-related acute kidney injury, dehydration or electrolyte imbalance related to dehydration	40% of AKI and dehydration admissions were considered possibly preventable if pharmacotherapy of high-risk medications (diuretic, ACE-I, ARB, NSAID or metformin) had been timely and adequately adjusted	N/A
Dietary nitrate supplementation does not influence thermoregulatory or cardiovascular strain in older individuals during severe ambient heat stress (2020) Cramer MN; Hieda M; Huang M; Moralez G; Crandall CG	Randomized crossover study	N/A	N/A	Results indicate that dietary nitrate supplementation as a possible intervention is NOT effective at reducing thermoregulatory or cardiovascular strain during heat stress in older adults
Risk factors for heat related death during the August 2003 heat wave in Paris, France, in patients evaluated at the emergency department of the Hôpital Européen Georges Pompidou (2006) Davido, A.; Patzak, A.; Dart, T.; Sadier, M.P.; Méraud, P.; Masmoudi, R.; Sembach, N.; Cao, T.H.	Retrospective observational study	Higher degree of dependent living was one risk factor associated with short-term mortality (78.3% of non-survivors had total or partial dependence)	Survivors were less often on psychotropic medications than non-survivors, but the difference for patients on diuretics or cardiotropes was not significant	N/A
Heat Illness Requiring Emergency Care for People Experiencing Homelessness: A Case Study Series (2022) English, T.; Larkin, M.; Vasquez Hernandez, A.; Hutton, J.; Currie, J.	Case series	Case One was 66 years old with multiple comorbidities and was ultimately diagnosed with heatstroke	Medications may have impaired thermoregulation in Case One (benzatropine, amisulpride, amlodipine + olmesartan, and aclidinium)	N/A
Low-dose ASA therapy does not alter core or skin temperature during hot-dry or warm-humid heat stress (PSU HEAT project) (2025) Fisher KG; Leach OK; Cottle RM; Alexander LM; Kenney WL	Randomized, double-blind, placebo-controlled crossover study	Study participants were 66-80 years old	Low-dose aspirin did NOT alter core or skin temperatures in either the warm-humid or hot-dry environments, but it did reduce skin blood flow responses to heat stress	N/A
The effect of the summer 2003 heat wave on mortality in the Netherlands (2005) Garssen, J.; Harmsen, C.; de Beer, J.	Descriptive report	Strong association between increasing age and excess mortality (0-64 years r^2=0.16, 65-79 years r^2=0.43, 80+ years r^2=0.65); impact of heat wave was most pronounced among institutionalized older adults	N/A	N/A
Chronic statin therapy is associated with enhanced cutaneous vascular responsiveness to sympathetic outflow during passive heat stress (2019) Greaney JL; Stanhewicz AE; Kenney WL	Prospective cross-sectional physiological study	Results indicate that older adults with hypercholesterolemia have impaired reflex cutaneous vasodilation	N/A	Results indicate that chronic statin treatment may improve reflex cutaneous vasodilatation during heat stress

The effect of ambient temperature on type-2-diabetes: case-crossover analysis of 4+ million GP consultations across England (2017)<br>Hajat S; Haines A; Sarran C; Sharma A; Bates C; Fleming L	Case-crossover study	Patients aged 65+ seemed to be at greater risk for heat-related GP consultation, but this was not statistically significant	NO association between heat-related consultations and diuretics, anticholinergics, antipsychotics or antidepressants	N/A
Risk factors for hyperthermia mortality among emergency department patients (2021)<br>Hall C; Ha S; Yen IH; Goldman-Mellor S	Case-control study	Mean age for hyperthermia mortality cases was only 56; cases had statistically significant higher odds of past ED utilization for alcohol use, having Medicare, or having no insurance	N/A	N/A
Prognostic factors in non-exertional heatstroke (2010)<br>Hausfater, P.; Megarbane, B.; Dautheville, S.; Patzak, A.; Andronikof, M.; Santin, A.; André, S.; Korchia, L.; Terbaoui, N.; Kierzek, G.; Doumenc, B.; Leroy, C.; Riou, B.	Cohort study	Living in an institution, age >80 years, cardiac disease, and cancer were among the 9 independent prognostic factors; notably, 35% of non-survivors were autonomous prior to hospitalization	Chronic treatment with diuretics was among the 9 independent prognostic factors	N/A
Serum sodium abnormalities during nonexertional heatstroke: incidence and prognostic values (2012)<br>Hausfater, P; Mégarbane, B; Fabricatore, L; Dautheville, S; Patzak, A; Andronikof, M; Santin, A; Kierzek, G; Doumenc, B; Leroy, C; Manamani, J; Peviriéri, F; Riou, B	Cohort study (ancillary study to Hausfater 2010)	Mean age of admitted hyperthermic patients was 82 ±15; dementia and living in an institution were among the independent variables associated with hypernatremia; heatstroke severity score was one variable associated with hyponatremia	No statistically significant results	N/A
Urinary incontinence, hyperthermia, and sudden death (2011)<br>Herbst J; Gilbert JD; Byard RW	Case report	84 year old woman died due to strenuous exercise on an extremely hot day	Authors suggest that anticholinergic medication (oxybutynin for urinary incontinence) likely exacerbated the effects of extreme heat	N/A
Summer syncope syndrome redux (2015)<br>Huang JJ; Desai C; Singh N; Sharda N; Fernandes A; Riaz IB; Alpert JS	Case-control study (extension of Huang 2014)	Patients aged 60+ appeared to be the most vulnerable for developing syncope and cases of syncope were more frequent in the summer months	Syncope cases were more frequent in the summer months among those taking antihypertensive medications, but no specific drug class had a greater association; among taking antihypertensives, there was a significant increase in cases of syncope secondary to dehydration during the summer months (40.5%) vs winter months (29%)	N/A
Summer syncope syndrome (2014)<br>Huang JJ; Sharda N; Riaz IB; Alpert JS	Case-control study	Patients aged 60+ appeared to be the most vulnerable for developing syncope and cases of syncope were more frequent in the summer months	Syncope cases were more frequent in the summer months among those taking antihypertensives; antihypertensive medications may excessively lower blood pressure in hot and dry climates, leading to more frequent syncopal episodes and dehydration	N/A
The impact of the 2003 heat wave on daily mortality in England and Wales and the use of rapid weekly mortality estimates (2005)<br>Johnson, H.; Kovats, R.S.; McGregor, G.; Stedman, J.; Gibbs, M.; Walton, H.	Descriptive report	Excess mortality was greatest in the 75+ age group: 22% (CI: 20% to 25%) in England and Wales and 59% (CI: 51% to 67%) in London specifically	N/A	N/A
Increased Risk of Drug-Induced Hyponatremia during High Temperatures (2017)<br>Jönsson AK; Lövborg H; Lohr W; Ekman B; Rocklöv J	Case-crossover study	Median patient age for drug-induced hyponatremia reports was 80 years	Increasing odds ratio with increasing temperature during warm months; the change in sodium per 1 degree C increase in temperature was estimated to be -0.37 mmol/L	N/A
Syndromic surveillance and heat wave morbidity: a pilot study based on emergency departments in France (2009)	Retrospective observational study	The proportion of emergency department visits related to heat wave disease syndromes (HWDS) significantly increased during 'on	N/A	N/A

Josseran, L.; Caillère, N.; Brun-Ney, D.; Rottner, J.; Filleul, L.; Brucker, G.; Astagneau, P.		alert' period (ONAP) for older adults		
Increased risk of hospital admission for dehydration or heat-related illness after initiation of medicines: a sequence symmetry analysis (2016)<br>Kalisch Ellett LM; Pratt NL; Le Blanc VT; Westaway K; Roughead EE	Sequence symmetry analysis	Median age of dehydration or heat-related admissions during study period was 85 years	Increased relative risk upon initiation of anticoagulants, nitrates, diuretics, beta blockers, calcium channel blockers, ACEI, ARB, NSAIDs, antipsychotics, antidepressants, and anticholinergic agents; risk was highest for ACEI in combination with a diuretic (ASR 2:79, 95% CI 1:53-4:43); no significant association for anticonvulsants, anti-Parkinson's agents, hypnotics, anxiolytics, or antihistamines	N/A
The health impacts of heat waves in five regions of New South Wales, Australia: A case-only analysis (2010)<br>Khalaj, B.; Lloyd, G.; Sheppeard, V.; Dear, K.	Case-only study	Men aged 75+ were more likely to be hospitalized with a primary diagnosis of heat-related illness; patients aged 65+ were more likely to be hospitalized when their underlying condition was cerebrovascular disease	N/A	This study was part of a program on adaptation to climate change undertaken by the NSW state health department
The short-term influence of weather on daily mortality in congestive heart failure (2007)<br>Kolb, S.; Radon, K.; Valois, M.-F.; Héguy, L.; Goldberg, M.S.	Case-crossover study	Increased maximum daily temperature was associated with increased daily mortality in older patients with congestive heart failure	N/A	N/A
Implementation evaluation of the Dutch national heat plan among long-term care institutions in Amsterdam: a cross-sectional study (2013)<br>Kunst AE; Britstra R	Cross-sectional study	Identifies older adults as a vulnerable group during heat waves	Authors suggest that heat plans place greater emphasis on the use of medications that may increase risk of heat-related mortality, since only 41% considered consulting physicians about it to be "very important"	Assesses an existing heat plan to protect older adults during heat waves, including what strategies were actually implemented and what barriers exist to implementation
Prevalence of risk and protective factors associated with heat-related outcomes in Southern Quebec: A secondary analysis of the NuAge study (2015)<br>Laverdière É; Généreux M; Gaudreau P; Morais JA; Shatenstein B; Payette H	Cross-sectional study	Study population is older adults; the most prevalent risk factors were cardiovascular medication (50.8%), hypertension (46.7%), living alone (39.2%), cardiovascular disease (36.9%), living in an urban heat island (34.7%), and needing help in daily activities (26.5%)	Use of cardiovascular medication was the most prevalent risk factor in study population	N/A
Heatwaves, medications, and heat-related hospitalization in older Medicare beneficiaries with chronic conditions (2020)<br>Layton JB; Li W; Yuan J; Gilman JP; Horton DB; Setoguchi S	Case series analysis	Results suggest that the study population (older adults with chronic comorbidities taking heat-sensitizing medications) are at increased risk of heat-related hospitalization, even when the heat is not "extreme"	No statistically significant positive synergistic effects were found between heatwaves and medications, but several medications were associated with increased risk of heat-related hospitalization in the summer months in general (ACE inhibitors or ARBs, anticholinergic agents, antipsychotics, and loop diuretics)	N/A
No effect of stage 1 hypertension or hypertensive medication on critical environmental limits (PSU HEAT Project) (2025)<br>Leach, O.K.; Fisher, K.G.; Cottle, R.M.; Kenney, W.L.	Extension of Fisher 2025 (randomized, double-blind, placebo-controlled crossover study)	Stage 1 hypertension as a comorbidity did NOT affect critical environmental limits	Antihypertensive medication did NOT affect critical environmental limits	N/A
Psychotropic drugs use and risk of heat-related hospitalisation (2007)<br>Martin-Latry K; Goumy MP; Latry P; Gabinski C; Bégaud B; Faure I; Verdoux H	Matched case-control study	Mean age of patients presenting with heat-related pathologies was 83 years, and 32% were aged 90+	Anticholinergic drugs (OR 6.0, 95% CI 1.8-19.6), antipsychotics (OR 4.6, 95% CI 1.9-11.2) and anxiolytics (OR 2.4, 95% CI 1.3-4.4) were associated with admission	N/A

A retrospective study of seasonal variation in sodium-glucose co-transporter 2 inhibitor-related adverse events using the Japanese adverse drug event report database (2024) Matsumoto K; Goto F; Maezawa M; Nakao S; Miyasaka K; Hirofuji S; Shiota K; Ichihara N; Yamashita M; Nokura Y; Yamazaki T; Sugishita K; Tanaka H; Tamaki H; Ishiguro M; Iguchi K; Nakamura M	Retrospective observational	N/A	Higher incidence of dehydration-related adverse events for SGLT2 inhibitors during the summer months	N/A
The impact of the summer 2003 heat waves on mortality in four Italian cities (2005) Michelozzi, P.; de Donato, F.; Bisanti, L.; Russo, A.; Cadum, E.; DeMaria, M.; D'Ovidio, M.; Costa, G.; Perucci, C.A.	Descriptive report	Excess mortality was the greatest in the 75-84 and 85+ age groups	N/A	N/A
Adverse drug reactions in patients older than 70 years during the heat wave occurred in France in summer 2003: A study from the French Pharmaco Vigilance Database (2006) Michenot, F.; Sommet, A.; Bagheri, H.; Mestre-Lapeyre, M.; Montastruc, J.L.	Retrospective observational study	68 ADRs related to heat occurred in 2003 (27 possible, 27 plausible, 14 likely); ADRs were reported more frequently in women and patients 80+	ADRs reported during summer 2003 were most commonly metabolic (dehydration or electrolyte disturbances) and neuropsychiatric (confusion, falls, or coma) and the most common drugs involved were diuretics, ACE inhibitors, antidepressants (mostly SSRIs), PPIs, digoxin, benzodiazepines, oral hypoglycemics, and sartans	N/A
Mortality of patients with heatstroke admitted to intensive care units during the 2003 heat wave in France: A national multiple-center risk-factor study (2006) Misset, B.; De Jonghe, B.; Bastuji-Garin, S.; Gattolliat, O.; Boughrara, E.; Annane, D.; Hausfater, P.; Garrouste-Orgeas, M.; Carlet, J.	Retrospective risk-factor study	Mean age of heatstroke ICU patients was 67.2 ± 14.1 years	Diuretic use was associated with hospital death in univariate analysis, but not in multivariate analysis	N/A
An unwell patient with Parkinson's disease: Hyperpyrexia syndrome in a heatwave (2022) Mooney, E.; Smith, M.D.; Henderson, E.J.	Case report	Extremely hot weather may have precipitated hyperpyrexia syndrome in 82 year old patient with Parkinson's Disease	N/A	N/A
Electric fan use for cooling during hot weather: a biophysical modelling study (2021) Morris NB; Chaseling GK; English T; Gruss F; Maideen MFB; Capon A; Jay O	Biophysical model	Uses impaired sweating ability of older adults with and without anticholinergic medications to develop model	Uses impaired sweating ability of older adults with and without anticholinergic medications to develop model	Authors suggest revised simplified temperature thresholds for electric fan use in order to increase the number of days fans can be used as a cooling device instead of air conditioning, which contributes to greenhouse gas emissions
Outdoor temperature and survival benefit of empiric potassium in users of furosemide in US Medicaid enrollees: A cohort study (2019) Nam, Y.H.; Bilker, W.B.; Leonard, C.E.; Bell, M.L.; Hennessy, S.	Cohort study	N/A	Basis of study is that furosemide-induced potassium depletion may be worsened during extreme heat due to sweating	Results indicate an increasing survival benefit of empiric potassium among furosemide users as daily maximum temperature increases, providing a possible intervention to reduce excess mortality during extreme heat (96.0 all-cause mortality rate for potassium users and 105.8 for non-users)
Diabetes in the desert: what do patients know about the heat? (2010) Nassar AA; Childs RD; Boyle ME; Jameson KA; Fowke M; Waters KR; Hovan MJ; Cook CB	Cross-sectional study	Results indicate that patients with poor glycemic control are potentially at increased risk for dehydration during hot weather (diabetes may increase heat sensitivity as a comorbidity)	N/A	Results indicate that there are knowledge gaps among diabetic patients living in a hot climate and that more patient education is needed

Risk of death related to psychotropic drug use in older people during the European 2003 heatwave: a population-based case-control study (2009) Nordon C; Martin-Latry K; de Roquefeuil L; Latry P; Bégaud B; Falissard B; Rouillon F; Verdoux H	Case-control study	Patients were aged 70-100 years	Significant dose-response relationship between the number of drugs and risk of mortality; antidepressants (SSRIs only) and antipsychotics ("other" only) were associated with a 20% and 40% increased risk of death before heatwave; antidepressants and antipsychotics (all) were associated with a 70% and 110% increased risk of death during heat wave; anxiolytics/hypnotics were associated with a decreased risk before heatwave, but during heatwave benzodiazepines had no association and non-benzodiazepine anxiolytics/hypnotics were associated with increased risk	N/A
Effect of Levodopa on Heat Hypersensitivity and Complex Motor Parkinsonism (2024) Noyes, E.; Rajput, A.H.; Bocking, S.; Rajput, A.	Case report	Thermoregulatory dysfunction leading to heat intolerance is a feature of multiple system atrophy (MSA) dysautonomia, suggesting that older adults with MSA as a comorbidity are at increased risk of heat-related illness	N/A (levodopa improved symptoms)	N/A
Heat-related morbidity in patients with orthostatic hypotension and primary autonomic failure (2005) Pathak, A.; Lapeyre-Mestre, M.; Montastruc, J.-L.; Senard, J.-M.	Case-control study	NO statistically significant association between orthostatic hypotension (OH)-related events during the heatwave and age, but autonomic failure (AF) may be a significant comorbidity to consider	N/A	N/A
Electrolyte disorders and in-hospital mortality during prolonged heat periods: a cross-sectional analysis (2014) Pfortmueller CA; Funk GC; Leichtle AB; Fiedler GM; Schwarz C; Exadaktylos AK; Lindner G	Cross-sectional study	Age ≥80 years was an independent predictor of mortality, but there was NO significant association between extreme heat and incidence of electrolyte disorders in the elderly population	N/A	N/A
Heat illness: predictors of hospital admissions among emergency department visits-Georgia, 2002-2008 (2014) Pillai, S.K.; Noe, R.S.; Murphy, M.W.; Vaidyanathan, A.; Young, R.; Kieszak, S.; Freymann, G.; Smith, W.; Drenzek, C.; Lewis, L.; Wolkin, A.F	Retrospective observational study	The odds of admission vs ED discharge increased with age, with the highest odds ratio for patients aged 80+ (OR 10.90); several comorbidities also increased odds of admission	N/A	N/A
Summary of the mortality impact assessment of the 2003 heat wave in France (2005) Pirard, P.; Vandentorren, S.; Pascal, M.; Laaidi, K.; Le Tertre, A.; Cassadou, S.; Ledrans, M.	Descriptive report	The greatest excess mortality was observed in the group aged 75 years and older and 2852 deaths were directly heat-related	N/A	N/A
Severe Hyponatremia Is Often Drug Induced: 10-Year Results of a Prospective Pharmacovigilance Program (2019) Ramírez E; Rodríguez A; Queiruga J; García I; Díaz L; Martínez L; Muñoz R; Muñoz M; Tong HY; Martínez JC; Borobia AM; Carcas AJ; Frías J	Prospective evaluation	Significant association between drug-induced severe hyponatremia and age	Significant association between drug-induced severe hyponatremia and high environmental temperature; cardiovascular or nervous system drugs were most frequent; hydrochlorothiazide was the most common culprit	N/A
Is environmental temperature related to renal symptoms, serum lithium levels, and other laboratory test results in current lithium users? (2014) Rej S; AlAqeel B; Segal M; Low NC; Mucsi I; Holcroft C; Looper K	Cross-sectional study	N/A	NO significant association between renal parameters or other laboratory tests with temperature in lithium users	N/A

The combined effect of high ambient temperature and antihypertensive treatment on renal function in hospitalized elderly patients (2016) Sagy, I.; Vodonos, A.; Novack, V.; Rogachev, B.; Haviv, Y.S.; Barski, L.	Cohort study	Negative effect of temperature on kidney function was most noticeable in patients older than 75	Higher daily temperature is associated with impaired kidney function in older adults treated with antihypertensives (thiazides and ACE/ARBs)	N/A
Influence of Outdoor Temperature and Relative Humidity on Incidence and Etiology of Hyponatremia (2019) Sailer, CO; Winzeler, B; Nigro, N; Bernasconi, L; Mueller, B; Christ-Crain, M	Cross-sectional cohort study	Significant positive correlation between profound hyponatremia and outdoor temperature (1.2% increased risk per degree C); significant association between hyponatremia and age	Significant increase in diuretic-induced hyponatremia with increase in temperature (4% per degree C)	N/A
Seasonal variation in the prevalence of profound hyponatremia (<125 mmol/l) in patients on admission to an acute hospital in Japan (2019) Sasaki, N.; Aoki, Y.	Cross-sectional study	Results confirm susceptibility of older adults to hyponatremia and seasonality of hyponatremia	Authors suggest that diuretics may contribute to incidence of profound hyponatremia in warmer months, but NO statistically significant difference was found in this study	N/A
Susceptibility to heat wave-related mortality: a follow-up study of a cohort of elderly in Rome (2009) Schifano, P.; Cappai, G.; De Sario, M.; Michelozzi, P.; Marino, C.; Bargagli, A.M.; Perucci, C.A.	Cohort study	Age, being unmarried, and certain comorbidities were significant effect modifiers for mortality during heat waves (but the cause of death was not analyzed)	N/A	N/A
Heat-related cardiovascular morbidity and mortality in Switzerland: a clinical perspective (2021) Schulte F; Röösli M; Ragettli MS	Time-series analysis	More deaths occurred in patients 75 years and older	Use of antihypertensive medications may explain the heat-related mortality increase for hypertension and heart failure as underlying conditions, despite decreased hospital admissions for these conditions	N/A
Mortality in Spain during the heat waves of summer 2003 (2005) Simón, F.; Lopez-Abente, G.; Ballester, E.; Martínez, F.	Descriptive report	Excess mortality was observed in the 74-84 and 85+ age groups (15.08% and 28.76%)	N/A	N/A
A comparative study of adverse drug reactions during two heat waves that occurred in France in 2003 and 2006 (2012) Sommet, A.; Durrieu, G.; Lapeyre-Mestre, M.; Montastruc, J.-L.	Retrospective observational study	Study population was patients older than 70 years who experienced serious ADRs during heat wave or reference period	Significantly more ADRs were heat related in 2003 and 2006 (22.4% and 20.4%) than in reference period (11.0%); most frequently involved drugs during heat waves were diuretics, serotonic antidepressants, ACE inhibitors, and PPIs	N/A
Relationship between heat index and mortality of 6 major cities in Taiwan (2013) Sung, T.I.; Wu, P.C.; Lung, S.C.; Lin, C.Y.; Chen, M.J.; Su, H.J.	Time-series analysis	Mortality increased with daily mean heat index and with age	N/A	N/A
Prevalence, recurrence and seasonal variation of hyperkalemia among patients on hemodialysis (2022) Tsiagka, D.; Georgianos, P.I.; Pikilidou, M.I.; Vaios, V.; Roumeliotis, S.; Syrganis, C.; Mavromatidis, K.; Metallidis, S.; Liakopoulos, V.; Zebekakis, P.E.	Retrospective observational study	NO significant association between hyperkalemia (sK > 5.5 mM) and age was found, but there was a significant association between hyperkalemia and season	NO significant association between hyperkalemia and use of insulin, beta blockers, or RAS blockers	N/A
The effect of heat waves on ambulance attendances in Brisbane, Australia (2013) Turner, L.R.; Connell, D.; Tong, S.	Time-series analysis	The 65-74 and 75+ age groups were especially vulnerable to heat and had more total, respiratory, and cardiovascular ambulance attendances		N/A
The role of psychotropics on the associations between extreme temperature and heat-related outcomes among people with mental health conditions: population-based study (2024) Wong, A.Y.S.; Iwagami, M.; Taniguchi, Y.; Kawamura, C.; Suzuki, A.; Douglas, I.J.;	Self-controlled case series	N/A	No significant association between psychotropic drug use and increased risk of heat-related illness (there was a 40-60% increased risk of heat-related illness associated with heatwave in people with mental illness regardless of psychotropic drug use)	N/A

Bhaskaran, K.; Sugiyama, T.; Kuroda, N.; Nitsch, D.; Tamiya, N.				
Risk factors for direct heat-related hospitalization during the 2009 Adelaide heatwave: a case crossover study (2013) Zhang, Y.; Nitschke, M.; Bi, P.	Case-crossover study	Univariate analysis suggests age as significant risk factor for heat-related hospitalization, as well as living alone and use of community services (which may indicate restricted mobility)	N/A	N/A
Grey Literature				
Apipiprazole, NuCare Pharmaceuticals, Inc.; brexiprazole, Otsuka America Pharmaceutic, Inc.; cariprazine, Allergan, Inc.; dicyclomine HCl, Solaris Pharma Corporation; empagliflozin, Aphena Pharma Solutions - Tennessee, LLC; glycopyrrolate, Quagen Pharmaceuticals, LLC; glycopyrronium, Journey Medical Corporation; iloperidone, Vanda Pharmaceuticals Inc.; lumateperone, Intra-Cellular Therapies, Inc.; lurasidone, Direct_rx; olanzapine, Remedyrepack, Inc.; olanzapine and fluoxetine, Teva Pharmaceuticals USA, Inc.; olanzapine and samidorphan, Alkermes, Inc.; paliperidone, Major Pharmaceuticals; phentermine and topiramate, Prasco Laboratories; prochlorperazine, Stat Rx USA, LLC; quetiapine, H2-Pharma, LLC; risperidone, Indivior Inc.; sofpironium, Botanix SB Inc.; asenapine, Noven Therapeutics, LLC; scopolamine transdermal patch system, Padagis Israel Pharmaceuticals Ltd.; topiramate, Quallent Pharmaceuticals Health LLC; ziprasidone, NorthStar Rx LLC; zonisamide, Azurity Pharmaceuticals, Inc.	Prescribing information	N/A	Indicates risk of heat-related illness associated with this medication	Provides official drug information that warns of risk in order to inform prescribing practices and improve patient counseling
Heat response plans: summary of evidence and strategies for collaboration and implementation (2020) Jessica Abbinett, Paul J. Schramm, Stasia Widerynski, Shubhayu Saha, Suzanne Beavers, Margaret Eaglin, Uei Lei, Seema G. Nayak, Matthew Roach, Matt Wolff, Kathryn C. Conlon, Lauren Thie	Report	Identifies older adults as a vulnerable group	N/A	Summarizes evidence and strategies for heat response plans in order to inform future efforts to prevent heat-related morbidity and mortality
Extreme Heat Adaptation (2024) U.S. CDC	Webpage	N/A	N/A	Summarizes various heat adaptation activities implemented by CRSCI grant recipients in their jurisdictions to protect their communities from the health effects of climate change, including extreme heat
Impacts of Extreme Weather Conditions and Disasters on Older Adult Health (2024) NIA and diverse group of panelists	Virtual workshop	Vulnerability of older adults during climate change is the focus of the workshop; extreme heat is primarily discussed in Dr. Carr's presentation, "Global Population Aging and Heat Exposure in the 21st Century: Implications for Late-Life Well-Being and Policy"	Briefly mentioned in Dr. Carr's presentation	Shows that there is research being done and shared across a variety of disciplines to address the vulnerability of older adults during climate crises; Dr. Carr's presentation highlights the need for specific interventions that consider the interplay of population aging and rising temperatures
Climate change and extreme heat: what you can do to prepare (2016) U.S. CDC and EPA	Booklet	Identifies older adults as a vulnerable group	Mentioned briefly as a risk factor	Educates the general public on rising global temperatures, how it affects health, and what people can do before and during an extreme

				heat event to minimize health impact
People at Increased Risk for Heat-Related Illness (2024)<br>U.S. CDC	Webpage	Identifies older adults as a vulnerable group	N/A	Educates the general public by providing a brief overview of people who are susceptible to heat-related illness with links to additional resources
Heat and Older Adults (Aged 65+) (2024)<br>U.S. CDC	Webpage	Lists several reasons why older adults are at higher risk of heat-related illness	Lists as a risk factor	Educates older adults and caretakers on the risk of heat-related illness during extreme heat, including several preventative measures to take
Protect Yourself From the Dangers of Extreme Heat (2024)<br>U.S. CDC	Webpage	Identifies older adults as a vulnerable group	N/A	Educates the general public on heat-related illness prevention with attached infographics, fact sheets, social media graphics, and links to other resources
How to use the HeatRisk Tool and Air Quality Index (2024)<br>U.S. CDC	Webpage	N/A	N/A	Educates healthcare providers on the HeatRisk Tool and AQI and how to advise patients/caregivers on using these tools
Heat & Health Tracker<br>U.S. CDC	Interactive tool	Addresses the sensitivity of older adults and other at-risk groups to heat	N/A	Allows users to input their zipcode and see the heat and health data for their area at any time, so that they can stay safe on hot days and recognize when the weather may pose a risk to health
Clinical Overview of Heat (2024)<br>U.S. CDC	Webpage	Identifies older adults as a vulnerable group	Summarizes risk posed by some medications and links to Heat and Medications webpage	Educates healthcare providers on the effects of heat on health, suggests creating heat action plans with patients, and provides links to additional resources
Quick Start Guide for Clinicians on Heat and Health (2024)<br>U.S. CDC	Webpage	Acknowledges other relevant risk factors but does not explicitly identify older adults as at-risk	Lists medications as risk factor and suggests review of medications as part of Heat Action Plan	Provides clinicians with an outline of steps to take to protect patients during hot weather
CHILL'D-Out: A Heat and Health Risk Factor Screening Questionnaire<br>U.S. CDC	Webpage	Acknowledges other relevant risk factors but does not explicitly identify older adults as at-risk	Lists medications as a risk factor in the questionnaire	Allows healthcare providers to assess patients' risk factors from heat or poor air quality and suggests a Heat Action Plan
Heat and Medications - Guidance for Clinicians (2024)<br>U.S. CDC	Webpage	Recognizes high rates of medication use among older adults, leading to increased risk	Summarizes what is known about drug classes and heat risk	Provides guidance for clinicians on how to advise patients and possibly adjust medication regimens during heat waves
Heat-related health dangers for older adults soar during the summer (2018)<br>Calvin, Kim (NIA)	News release	Discusses lifestyle and underlying health factors of older adults that increase risk of hyperthermia	Identifies medications that reduce sweating and polypharmacy as risk factors	Published during the summer to educate older adults and caretakers on heat-related illness, risk factors, and safety tips
Advice for older adults on staying safe in hot weather (2017)<br>Calvin, Kim (NIA)	News release	Discusses lifestyle and underlying health factors of older adults that increase risk of hyperthermia	Identifies medications that reduce sweating and polypharmacy as risk factors	Published during the summer to educate older adults and caretakers on heat-related illness, risk factors, and safety tips
Hot Weather Safety for Older Adults (2022)<br>NIA	Webpage	Lists several risk factors that make older adults vulnerable during hot weather	Identifies prescription medication use as a risk factor	Provides the public with recommendations for each heat-related illness, list of safety tips, and list of external resources
Infographic: Staying Safe in Hot Weather (2025)<br>NIA	Infographic	Guidance is for older adults	N/A	Educates the public about signs of hyperthermia and safety tips; encourages distribution of infographic to reach wider audience

Table S3. Scientific Literature Characteristics

**Study Design**	
Cohort	10 (16.4%)
Retrospective observational	8 (13.1%)
Case report/series	8 (13.1%)
Cross-sectional	7 (11.5%)
Case-control	6 (9.83%)
Descriptive report	5 (8.20%)
Time series analysis	4 (6.56%)
Case-crossover	4 (6.56%)
Randomized crossover	3 (4.92%)
Sequence symmetry analysis	1 (1.64%)
Prospective evaluation	1 (1.64%)
Case-only	1 (1.64%)
Cross-sectional cohort	1 (1.64%)
Retrospective risk-factor	1 (1.64%)
Biophysical model	1 (1.64%)
**Location**	
United States	14 (23.0%)
France	11 (18.0%)
Australia	9 (14.8%)

Canada	4 (6.56%)
United Kingdom	3 (4.92%)
Japan	3 (4.92%)
Netherlands	3 (4.92%)
Switzerland	3 (4.92%)
China	2 (3.28%)
Israel	2 (3.28%)
Italy	2 (3.28%)
Spain	2 (3.28%)
Greece	1 (1.64%)
Sweden	1 (1.64%)
Taiwan	1 (1.64%)
Total	61 (100%)

Table S4. Grey Literature Characteristics

Origin	N (%)
U.S. Centers for Disease Control and Prevention (CDC) Climate and Health website	12 (29.3%)
National Institute on Aging (NIA) website	5 (12.2%)
U.S. Food and Drug Administration (FDA) Label database	24 (58.5%)
TOTAL	41 (100%)
**Format**	**N (%)**
Webpage	10 (24.4%)
Interactive tool	1 (2.44%)
News release	2 (4.88%)
Virtual workshop	1 (2.44%)
Infographic	1 (2.44%)
Prescribing information	24 (58.5%)
Report	1 (2.44%)
Pamphlet	1 (2.44%)
TOTAL	41 (100%)
**Primary Intended Audience**	**N (%)**
Patients or general public	10 (24.4%)
Healthcare professionals or prescribers	29 (70.7%)
Public health officials	1 (2.44%)
Researchers	1 (2.44%)
TOTAL	41 (100%)
